# Transcranial direct current stimulation neuromodulates intracranial cognitive evoked activity in humans

**DOI:** 10.1073/pnas.2416541122

**Published:** 2025-06-03

**Authors:** Mireille Tabikh, Tom Quetu, Louis Maillard, Sophie Colnat-Coulbois, Bruno Rossion, Laurent Koessler

**Affiliations:** ^a^Ingenierie Moleculaire, Cellulaire et Physiopathologie, UMR7365, CNRS and University of Lorraine, Nancy F-54000, France; ^b^Epilepsy surgery unit, Neurology Department, University Hospital of Nancy, Nancy F-54000, France; ^c^Epilepsy surgery unit, Neurosurgery Department, University Hospital of Nancy, Nancy F-54000, France; ^d^Bioserenity, Paris F-75013, France

**Keywords:** transcranial direct current stimulation, neuromodulation, human in vivo, intracranial EEG recording, cognitive evoked activity

## Abstract

Transcranial direct current stimulation (tDCS) is an easy to use, noninvasive brain stimulation technique that gained prominence for its potential in cognitive rehabilitation. Electroencephalography (EEG), which records electrical brain activity with a high temporal resolution, is well suited to quantify tDCS-induced neuromodulation in humans. However, most studies relying on scalp EEG recordings or event-related potentials showed low reliability and only indirect correlations. Here, we combined intracranial EEG (iEEG) recordings with a sham-controlled tDCS experiment during fast periodic visual stimulation. Anodal (+2 mA) tDCS was applied over the right occipito-temporal cortex for 20 min using two small ring high-definition electrodes. Through the analysis of iEEG signals of 947 intracerebral contacts in 11 drug-resistant epileptic patients, we quantified the neuromodulation of iEEG cognitive evoked responses during (P2 phase) and after (P3 phase) tDCS by comparison to a control phase before tDCS (P1 phase). Significant neuromodulations of face-selective iEEG activity in anterior & posterior temporal lobe and in the occipital lobe were found, with amplitude increases of 3% and 4%, 16% and 13%, and 36% and 33%, during and after tDCS, respectively. Interestingly, despite a unique tDCS session, the face-selective neuromodulation in the right visual occipito-temporal cortex remained significant (*P* = 0.015) after tDCS (P3 vs. P1). This iEEG study demonstrates that using low intensity tDCS and small ring electrodes can induce significant electrophysiological effects on a selective cognitive function in humans.

## Significance

A sham-controlled transcranial direct current stimulation (tDCS) experiment was performed simultaneously with intracranial electroencephalographic recording (iEEG) and fast periodic visual stimulation (FPVS) in epileptic patients. General visual and face-selective iEEG responses evoked by FPVS were quantified before, during, and after a single session of anodal tDCS performed over the right posterior temporal lobe (PTL) using small ring electrodes. Significant amplitude increases of face-selective iEEG responses were observed during tDCS, whereas weak neuromodulation occurred on general visual responses. The neuromodulations were observed in the PTL. Finally, and interestingly, the neuromodulation remained after a single tDCS session. This study shows that objective, selective, and significant neuromodulation can be obtained using small electrodes and low intensity with tDCS.

The development of noninvasive brain stimulation (NIBS) techniques has opened new avenues for cognitive rehabilitation in neurological and psychiatric diseases. Among these techniques, transcranial direct current stimulation (tDCS) stands out from other techniques for its potential to modulate neural activity using low-intensity currents and its ability to be used at home with very little inexpensive equipment (1). tDCS is based on injecting a low-intensity direct current (few milliamps) onto the scalp’s surface, for several minutes in one or several sessions, using two or more scalp electrodes (for recent review see ref. 2). Since the 2000s, the use of tDCS as an alternative (or complementary) treatment for different neurological, psychiatric, and cognitive disorders has increased exponentially [for review see ref. 3 in neurological disease, (4) in psychiatric disease].

Despite its promising effects in humans, tDCS effects have long been questioned due to the lack of evidence demonstrating the current’s ability to pass through the highly resistive skull and to reach brain tissue (5–7). Recently, human in vivo studies using low-intensity NIBS simultaneously combined with intracranial electroencephalographic (iEEG) recordings have shown that significant electric fields (EF) can reach cortical brain structures as well as deep medial brain structures (such as the hippocampi, cingulate gyri, and the amygdalae) (8). The current’s propagation from scalp to cortical brain structures in humans is one of the fundamental steps needed to obtain an electrophysiological modulation of brain activity. The second step, that has yet to be investigated, concerns the low-intensity current’s ability to modulate the electrophysiological cortical activity in the human brain supporting cognitive functions.

At this level, the precise quantification of electrophysiological modulation due to tDCS remains unknown in humans. This lack of electrophysiological evidence supports interpretation of tDCS effects as placebo or indirect (e.g., metabolic) effects. Objective electrophysiological quantifications have never been performed previously due to methodological constraints, especially the scarcity of access to iEEG recordings, the only reference method for direct recording of cortical brain sources with high spatial and temporal resolution. A number of previous studies combined scalp EEG recordings with tDCS during cognitive tasks, but several methodological drawbacks led to controversial results (9, 10). In particular, nonphysiological artifacts [different to physiological artifacts i.e., interaction between the stimulation and the biological tissues (11)] on scalp EEG signals during tDCS induce challenging denoising processes (12, 13) and unprecise characterization of the electrophysiological effects (14, 15).

Here, we circumvent these issues by simultaneously combining sham-controlled tDCS with iEEG recording during fast periodic visual stimulation (FPVS). FPVS, of “frequency-tagging,” is an approach that generates electrophysiological activity responses at known periodic frequencies with high signal-to-noise ratio (SNR), objectivity, and robust test–retest results (16, 17). Since the neural activity of interest can be confined to small (known) frequency bins, it is largely immune to tDCS artifacts (*SI Appendix*, Fig. S1), making this approach particularly well-suited to perform an intracerebral evaluation of neuromodulation characteristics of the periodically evoked iEEG responses (in terms of amplitude, SNR, and spatial distribution). In the present study, the noise amplitude remains stable (i.e., no increase between P1, P2, and P3 phases) and weak (2 to 3 times less than the signal amplitude) during tDCS.

iEEG recordings were obtained through minimally invasive procedure of intracerebral implantation, which does not significantly alter the volume conduction of the head [no current leakage; (18)], offers the ability to investigate electrophysiological neuromodulation during tDCS without artifacts in iEEG signals. In addition, iEEG permits to investigate deep, intermediate, and superficial cortical brain sources which is relevant to sampling large cognitive networks. In the present study, the ventral occipito-temporal cortex (VOTC) was investigated as it contains a highly functional and distributed network that is often explored in epileptic patients (19).

The well-validated FPVS paradigm used here is based on presenting nonface images of different object categories (e.g., animals, plants, houses, …) at a rapid rate of six images per second, i.e., at a frequency of 6 Hz. Images of human faces are inserted every 5th image (i.e., 6 Hz/5 = 1.2 Hz). The presentation of (nonface and face) images at 6 Hz elicits neural responses in the cerebral cortex at the same frequency as image presentation (i.e., at 6 Hz and harmonics: 12 Hz, 18 Hz, etc.) called general visual responses. Furthermore, if face images reliably elicit differential responses, these face-selective responses appear at 1.2 Hz (and harmonics: 2.4 Hz, 3.6 Hz, etc.) (19–21). Due to the periodicity of the FPVS, both types of evoked responses (general visual and face-selective) can be quantified in the frequency domain of the iEEG recording (20). Each FPVS phase contained two successive FPVS sessions (three phases with two sessions each i.e., six FPVS sessions) and each phase was performed before (sham; Phase P1), during (Phase P2), and after (sham; Phase P3) tDCS.

The main objective of this study was to quantify the intracranial electrophysiological effects induced by anodal (+2 mA) tDCS, performed over the right occipito-temporal lobe, on both general visual and face-selective iEEG evoked responses. Then, the issue of anatomical tDCS specificity was investigated by quantifying the intracranial electrophysiological effects in the three main anatomo-functional brain regions of the VOTC.

## Results

**Fig. 1.**
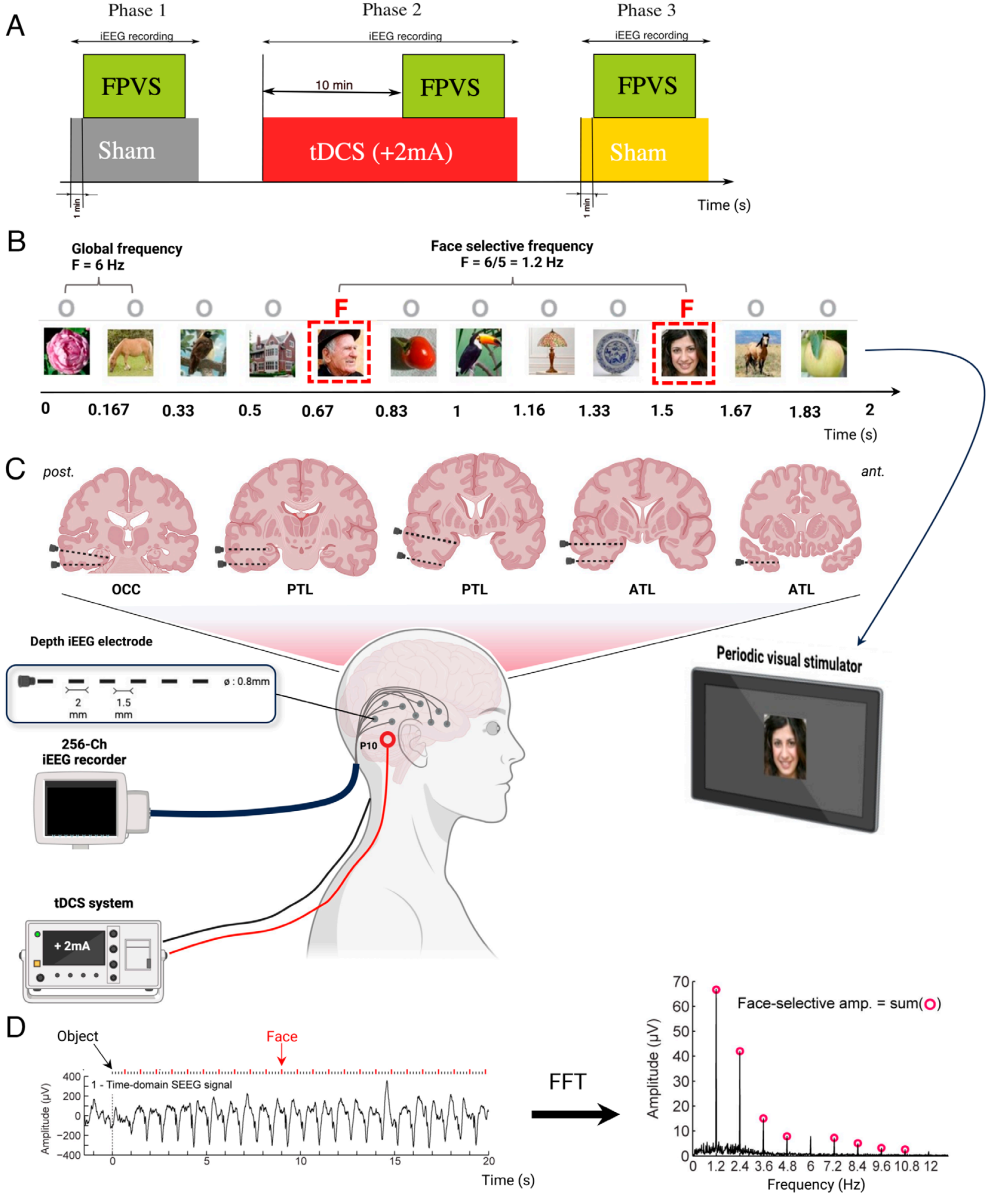
Overview of the combined iEEG-FPVS-tDCS protocol. (*A*) Timeline of the experiment with the three different phases of FPVS-iEEG sessions performed before, during, and after tDCS. (*B*) The FPVS paradigm. Images of objects were presented by sinusoidal contrast modulation at a rate of six stimuli per second (6 Hz). In the periodic condition, a different face image was presented every five stimuli (i.e., appearing at the frequency of 6/5 = 1.2 Hz). (*C*) Schematic view of the patient’s setting during the protocol. An average of eight iEEG multicontact electrodes were implanted in the VOTC from posterior (OCC) to anterior regions (ATL). (*D*) iEEG signal analysis. FFT was applied to iEEG signals in the time domain, with amplitude and SNR spectra extracted for all iEEG contacts. Here, face-selective and general visual amplitude responses (peaks) can be seen at 1.2 Hz and its harmonics (red dots) and 6 Hz plus its harmonic (12 Hz), respectively. Thanks to the duration of each FVPS session (71 s), the frequency resolution of the signal analysis is very high (i.e., 0.0141 Hz). iEEG; intracranial electroencephalography; FPVS: fast periodic visual stimulation; OCC: occipital; PTL: posterior temporal lobe; ATL: anterior temporal lobe; VOTC: ventral occipito-temporal cortex; FFT: Fast Fourier Transform.

Sham-controlled tDCS experiments were prospectively performed in 11 drug-resistant epileptic patients. The experiment contained three consecutive phases of iEEG recordings: P1 with sham tDCS during FPVS; P2 with +2 mA tDCS applied over the right VOTC (P10 electrode position) for 10 min (resting state period), followed by 10 min of active tDCS during FPVS (i.e., 20 min of tDCS in all); P3 with sham tDCS during FPVS. Active tDCS was blinded to the patients (Fig. 1*A*). FVPS in this paradigm elicits two types of responses: a general visual response, at 6 Hz and its harmonics, common to both face and nonface images, and a face-selective response, at 1.2 Hz and its harmonics, which reflects a response specific to face stimuli (Fig. 1 *B*–*D*). Patients’ attention before, during, and after tDCS remained optimal showing no significant changes in either response accuracies (100% in P1, P2, P3) and response times (mean values for P1: 0.46 s, P2: 0.48 s, P3: 0.47 s; *P* = 0.35).

In our population, a total of 90 intracranial electrodes were implanted in the right hemisphere, each electrode containing 5 to 18 contacts for independent recording of the local field potential (947 contacts in total). Then, iEEG contacts within the right hemisphere (where face-selectivity is the predominant; 21, 22) were investigated. All iEEG contacts in this study were selected because they recorded significant iEEG evoked responses during previous FPVS sessions (same cognitive paradigm i.e., objects and faces) performed during the week of patients’ iEEG investigation.

### TDCS Significantly Neuromodulates Face Selective Responses.

**Fig. 2.**
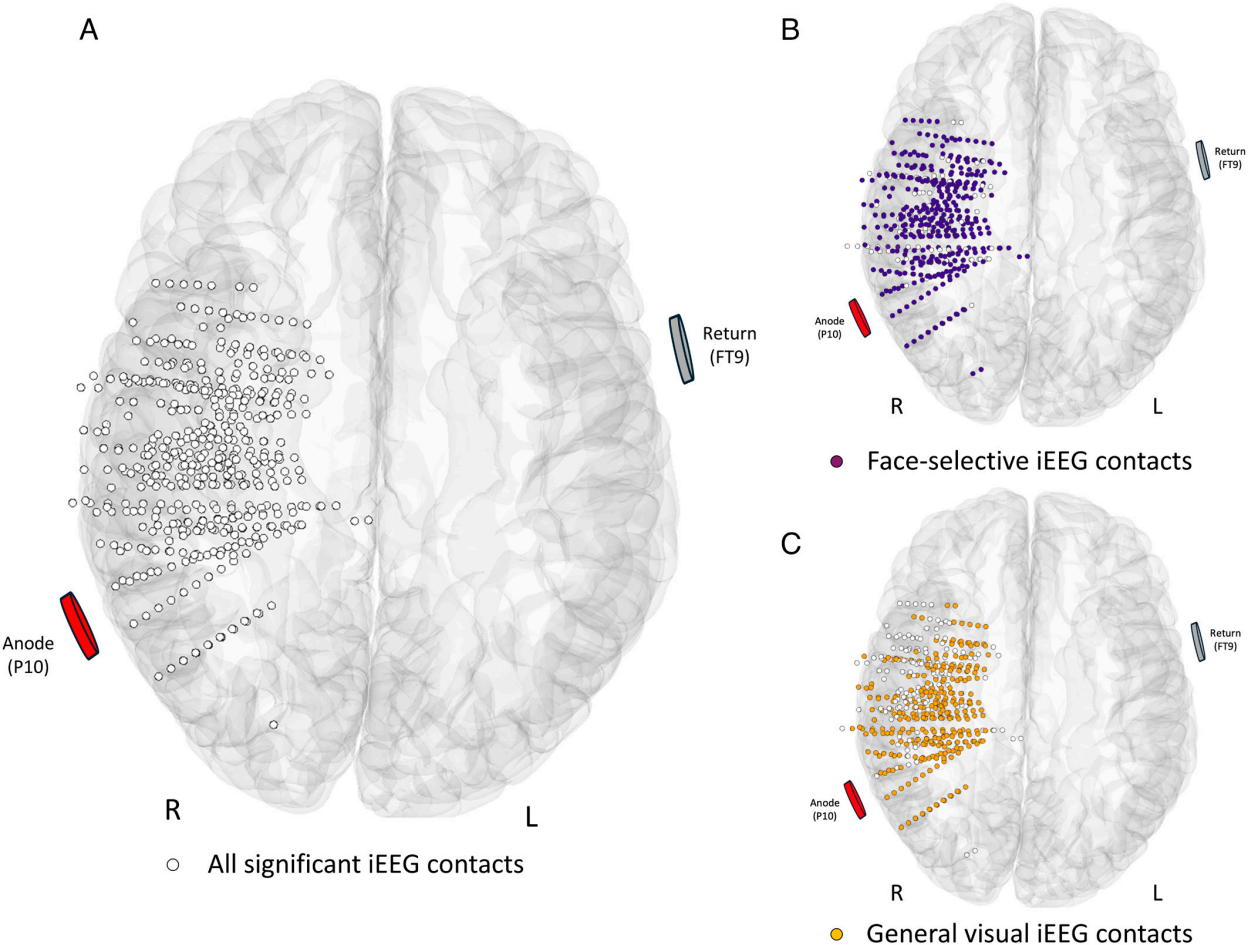
Spatial distribution of the iEEG contacts and the HD-ring tDCS electrodes in the MNI space (ventral view). (*A*) Maps of all significant iEEG recording contacts (n = 452) in the right hemisphere across the 11 individual brains displayed in the MNI space using transparent reconstructed cortical surface of the Colin27 brain. Each circle represents a single iEEG contact. For visualization purposes, individual contacts are displayed larger than their actual size (2 mm in length). (*B* and *C*) The same display for the iEEG contacts showing face-selective (n = 302; violet circles) and general visual (n = 260; orange circles) responses, respectively.

Among 453 iEEG contacts in the right hemisphere (Fig. 2*A*), 260 and 302 iEEG contacts which showed significant general visual and face-selective iEEG responses were used to compute the means and the SEM of these two kinds of neural responses, respectively (Fig. 2 *B* and *C*).

**Fig. 3.**
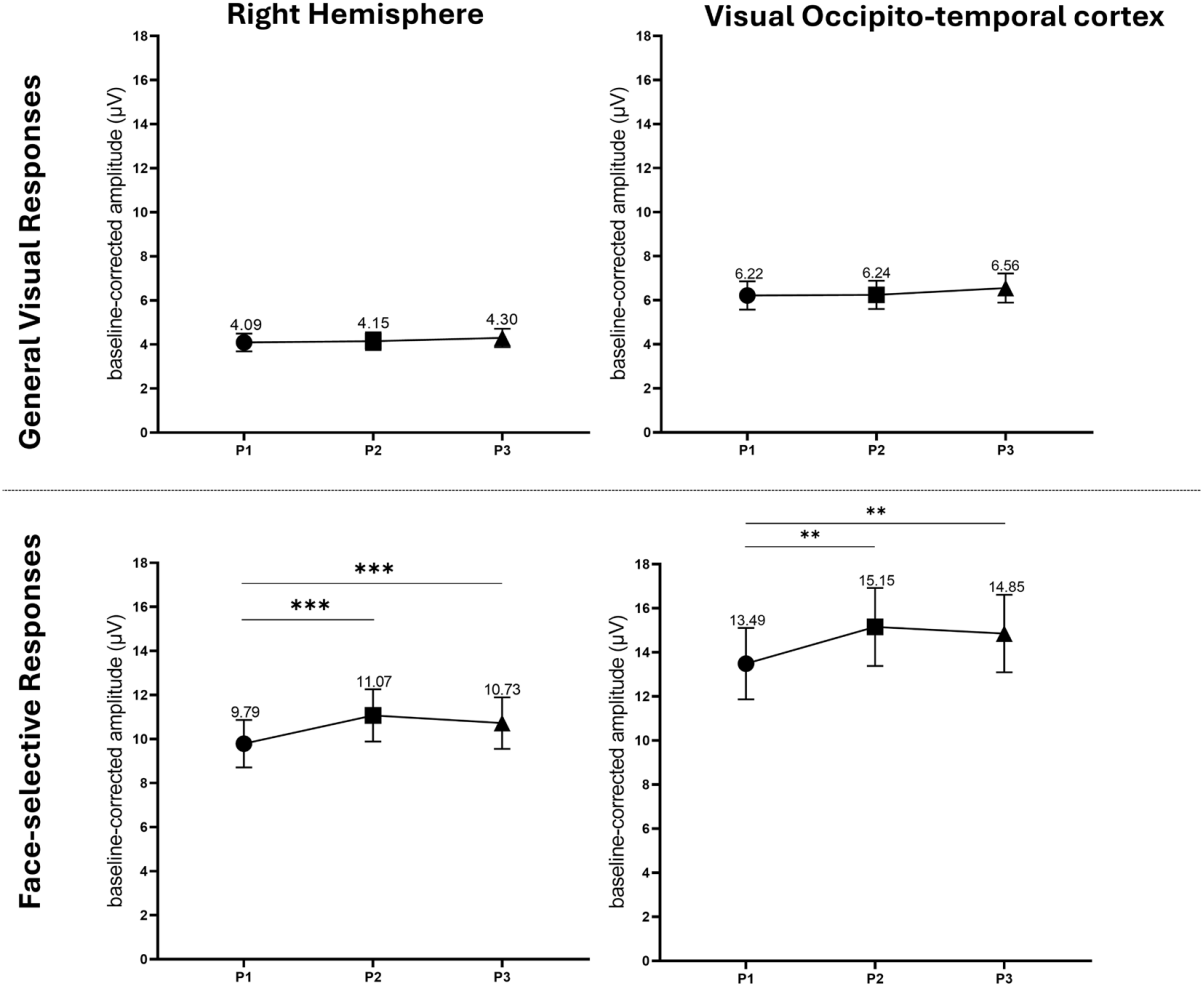
Neuromodulation of iEEG general visual (on the *Top*) and face-selective (on the *Bottom*) responses across the three phases of the tDCS experiment (before, P1; during, P2; after, P3) in the right hemisphere (*Left* column) and in the right visual occipito-temporal cortex (*Right* column). Averaged baseline-corrected amplitudes and SE of the mean of the iEEG evoked responses were computed during the three different phases for the 11 patients. In the right hemisphere, 260 and 302 iEEG contacts were used to compute the average baseline-corrected amplitudes whereas 147 and 181 contacts were selected for the general visual responses and the face-selective responses, respectively. Face-selective responses were significantly neuromodulated during (P2) and after (P3) tDCS (*P* < 0.001). In contrast, no significant neuromodulation was observed for the general visual responses. Graphs for SNR analysis are presented in *SI Appendix*, Fig. S2. Phases: Black circles: P1 (sham, before tDCS); Black squares: P2 (active, during tDCS); Black triangles: P3 (sham, after tDCS).

For general visual responses, the averaged baseline-corrected amplitudes and SEM recorded during phases P1, P2, and P3 were 4.09 ± 0.40, 4.15 ± 0.40, and 4.30 ± 0.41 µV, respectively. No significant baseline-corrected amplitude difference was found between the different phases (Fig. 3). The SNR values were 3.85, 3.78, and 3.93 during P1, P2, and P3, respectively (*SI Appendix*, Fig. S2).

For face-selective responses, the average baseline-corrected amplitudes recorded during the three phases (before, during, and after tDCS) were 9.79 ± 1.07, 11.07 ± 1.19 (i.e., +13%), and 10.73 ± 1.17 µV (i.e., +10%), respectively. A significant difference was found between P2 and P1 (*P* < 0.001) and between P3 and P1 (*P* < 0.001, Repeated measures ANOVA) (Fig. 3). The SNR values were 3.28, 3.37, and 3.49 during P1, P2, and P3, respectively (*SI Appendix*, Fig. S2).

Then, a more restrained iEEG analysis was performed using only the iEEG contacts located in the right VOTC.

For general visual responses, the average baseline-corrected amplitudes recorded from 147 contacts during phases P1, P2, and P3 were 6.22 ± 0.64, 6.24 ± 0.64, and 6.56 ± 0.69 µV, respectively. No significant baseline-corrected amplitude difference was observed between the different phases. The SNR values during the three phases were 4.91, 4.66, and 4.93, respectively.

For face-selective visual responses, the average baseline-corrected amplitudes recorded from 181 contacts during phases P1, P2, and P3 were 13.49 ± 1.62, 15.15 ± 1.77 (i.e., +12% with respect to P1), and 14.85 ± 1.76 µV (i.e., +10% with respect to P1), respectively. A significant baseline-corrected amplitude difference was found between P2 and P1 (*P* = 0.002) and between P3 and P1. (*P* = 0.003, Repeated measures ANOVA). The values of the SNR during the three respective phases were 3.83, 3.84, and 3.88, respectively (Fig. 3 and *SI Appendix*, Fig. S2).

### HD Electrodes Spatially Targeted Posterior Temporal Lobe (PTL).

**Fig. 4.**
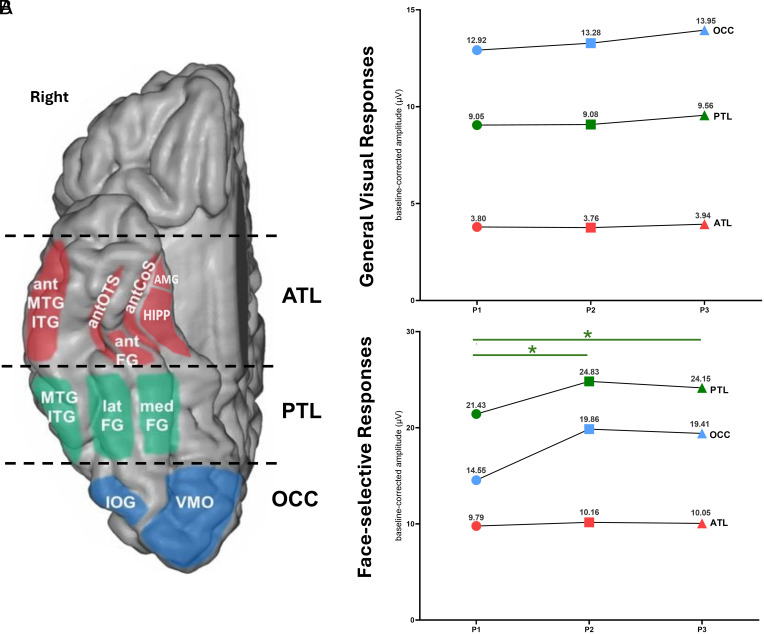
Neuromodulation of iEEG general visual and face-selective in the right VOTC. (*A*) Schematic anatomical view of each brain region of interest in the right VOTC. Brain regions are depicted over the right ventral surface of the Colin27 brain, although the iEEG responses were recorded by intracerebral contacts inside these regions. Three main regions (ATL in red, PTL in green, and OCC in blue) were defined to compare the spatial neuromodulation. (*B*) Baseline corrected amplitudes (mean; µV) of the general visual and the face-selective iEEG responses in the three ROIs during the three phases. A significant difference in the baseline-corrected amplitudes of the iEEG responses was observed in the PTL during the tDCS (P2) and maintained afterward (P3) in comparison to before tDCS (P1) (*P* = 0.003 & *P* = 0.015, respectively; repeated measure ANOVA). VOTC: ventral occipito-temporal cortex, ATL: Anterior Temporal Lobe, PTL: Posterior Temporal Lobe, OCC: Occipital Lobe. Colored circles: P1 (sham, before tDCS); colored squares: P2 (active, during tDCS); colored triangles: P3 (sham, after tDCS).

To investigate the spatial targeting of tDCS, 328 iEEG contacts in the VOTC for both iEEG responses were classified into three brain regions of interest (ROI): anterior temporal lobe (ATL), PTL, and occipital lobe (OCC) (Fig. 4*A*). This anatomical classification was previously defined in a previous iEEG study of the face-selective brain regions (21). The average EF values were respectively 0.29 ± 0.36 V/m, 0.17 ± 0.11 V/m and 0.22 ± 0.24 V/m in OCC, PTL, and ATL, respectively. In the inferior occipital gyrus (IOG), the average EF values were 0.38 ± 0.43 V/m (*SI Appendix*, Table S5). The baseline-corrected amplitudes and SNR of the iEEG evoked responses (both general visual and face-selective responses) located in each ROI were computed in each of the three phases of this study.

For general visual responses, the baseline-corrected amplitudes for the iEEG contacts located in the ATL (n = 91) were 3.8 ± 0.47, 3.76 ± 0.43, and 3.94 ± 0.44 µV during the three phases P1, P2, and P3, respectively. In the PTL (n = 40), the values were 9.05 ± 1.40, 9.08 ± 1.37, and 9.56 ± 1.44 µV, during the three phases P1, P2, and P3, respectively. Finally, the highest iEEG evoked responses were found in the OCC (n = 16) with 12.92 ± 3.09, 13.28 ± 3.34, and 13.95 ± 3.36 µV during the P1, P2, and P3, respectively. Despite an increasing trend, no significant change was found between the difference phases (Fig. 4*B*). The mean SNR values of the general visual responses were 3.65, 3.53, and 3.66 in the ATL, 5.96, 5.41, and 5.88 in the PTL, and 9.40, 9.19, and 9.80 in the OCC for phases P1, P2, and P3, respectively (*SI Appendix*, Fig. S3).

For face-selective responses, the baseline-corrected amplitudes for the iEEG contacts located in the ATL (n = 114) were 9.79 ± 1.20, 10.16 ± 1.23 (i.e., +4% with respect to P1) and 10.05 ± 1.22 µV (i.e., +3% with respect to P1), during the three phases P1, P2, and P3, respectively (no significant amplitude change). At the opposite, in the PTL (n = 51), the baseline corrected amplitudes were 21.43 ± 4.82, 24.83 ± 5.14 (i.e., +16% with respect to P1) and 24.15 ± 5.14 µV (i.e., +13% with respect to P1) during the three phases, respectively. The amplitude of the face-selective responses significantly varied from P1 to P2 i.e., during the tDCS (*P* = 0.003), as well as from P1 to P3 i.e., after the tDCS (*P* = 0.015) (Fig. 4*B*). In the OCC (n = 16), the baseline corrected amplitudes were 14.55 ± 3.56, 19.86 ± 5.41 (i.e., +36% with respect to P1), and 19.41 ± 5.24 µV (i.e., +33% with respect to P1) during the three phases, respectively. The variation of the amplitudes between P2 and P1 and between P3 and P1 showed a trend toward increase. The mean SNR values of the face-selective responses were 3.52, 3.55, and 3.48 in the ATL, 4.22, 4.32, and 4.44 in the PTL and 4.77, 4.36, and 4.91 in the OCC, during the three phases, respectively. (*SI Appendix*, Fig. S3).

## Discussion

### Significant Neuromodulation Using Low-Intensity, -Duration, and -Resolution TDCS Configuration.

Quantifying the intracerebral neuromodulation induced by NIBS in humans in vivo has not been performed before due to methodological and clinical constraints. Here, we overcame these constraints with simultaneous combination of tDCS with iEEG and FPVS. FVPS and iEEG were used as reference methods since they allow objective measurement of (frequency-tagged) brain activities directly within different brain sources organized in a sparse cognitive network and away from the stimulating scalp electrodes that generate electrical artifacts. In addition, the implementation of a sham-controlled experiment with iEEG recordings reduced placebo effects and allowed for a thorough investigation of the different tDCS phases (before, during, and after tDCS). The observed intracerebral neuromodulation was obtained in patients with preserved face perception abilities (23) and in cognitive brain networks similar to those previously mapped (21).

Despite setting tDCS parameters at a common intensity (2 mA), duration (20 min), and using a small number of electrodes (two small HD ring electrodes), tDCS significantly modulated the evoked iEEG responses. Deliberately, these parameters have not been optimized to study tDCS effects under the most frequent conditions encountered in research and clinical uses (24) and consequently, provide findings that would be broadly beneficial to the field. The quantification of iEEG neuromodulation in terms of amplitude showed significant but moderate increases (from 10 to 36%) which could be related to these routine tDCS parameters set in the study. Considering that neuromodulation is related to the intracerebral EF induced within the targeted brain network, one method to obtain a stronger EF, and consequently stronger neuromodulation, could be to increase tDCS intensity. In a combined NIBS-iEEG investigation, we demonstrate a direct linear relationship between the tDCS intensity and the measured intracerebral EF in humans. When tDCS intensity is doubled, the EF is also doubled (8). Yet, increasing the intensity is not without its constraints and dangers. For safety and tolerability considerations, increasing tDCS intensity (e.g., from 2 to 4 mA) can be facilitated by using larger electrodes like pads, which distribute the current over a broader area, or by employing a configuration of multiple small HD electrodes, which enhance focality (25). Considering the promising results of computational studies with high definition tDCS (26), this method should potentially achieve greater neuromodulation. Our original study which used small HD ring electrodes, instead of the more commonly used large sponges or pads, is also a first step in demonstrating that significant neuromodulation can be obtained using small ring electrodes despite the lack of an HD configuration. In the presurgical context of iEEG investigation, small HD electrodes are well suited to be positioned alongside the iEEG electrodes. This ergonomic aspect, along with their efficiency in distributing currents in the head tissues, is important to highlight, especially for broader clinical use.

Finally, the iEEG neuromodulation induced by tDCS in the posterior and OCC during our face-perception tasks could objectively explain the behavioral (27, 28) and scalp EEG (14, 15) changes and increases observed after tDCS in some studies.

### Significant Neuromodulation Remained After a Single-tDCS Session.

In this study, a single 20-min tDCS session was assigned for each participant. Despite this minimal procedure, interestingly, significant face-selective electrophysiological neuromodulation remained after the tDCS session. This finding confirms results showing that tDCS altered cortical excitability for approximately 1 h poststimulation, as demonstrated through scalp EEG evoked potential analysis (29, 30). Here, the iEEG aftereffects were measured immediately following the tDCS session (within a few minutes). The absence of longer investigation (e.g., 1 h or even more after the tDCS) was due to clinical reasons, specifically the removal of iEEG electrodes just after the experiment when clinical investigation was finished. Performing tDCS experiment earlier in the week is not feasible in the iEEG context because tDCS could disorganize the surrounding epileptic brain networks and therefore, bias the clinical diagnosis of epilepsy.

In a recent review (7), the main conclusion was the lack of evidence of cognitive effects in healthy populations (n = 59 analyses) resulting from a single session of tDCS. This apparent tDCS inefficacy should not be interpreted as a unique single-session issue because several other parameters such as the investigated brain cognitive networks (language, memory, executive functions, …), the control conditions, and the tDCS parameters (montage, duration, and intensity) were very different from one study to another and not fully controlled. Here, our finding using robust methodology, which provides electrophysiological evidence of tDCS effects, opens insights on the link between electrophysiological and behavioral neuromodulation. Indeed, the electrophysiological changes (i.e., the amplitude increases in the different brain regions of the VOTC) observed in our study are probably not strong enough to induce behavioral changes. Nevertheless, two important points must be made. First, the absence of behavioral changes after a single-session must not lead to the conclusion that tDCS is ineffective. Second, our results show that the low, or even null, cognitive effects could be primarily related to methodological aspects i.e., too moderate neuromodulation using common tDCS parameters.

For therapeutic purposes, more long-lasting aftereffects are desirable. Animal studies indicate that repeating DC stimulation during the aftereffects of an initial session enhances its efficacy (31, 32). Nonetheless, repeated induction of plasticity might also trigger homeostatic counteracting mechanisms (33). Two options were investigated in the literature to induce long-lasting effects. First, extending the duration of stimulation or increasing its intensity, both of which have been employed in clinical settings (34–36). The alternative method to extend tDCS aftereffects is through the repetition of tDCS sessions. Empirically, repetitive tDCS has been shown to produce cumulative effects when administered once daily in humans (37, 38).

This innovative combination of tDCS with iEEG recordings will be ideally suited to investigate this additional challenge i.e., the impact of tDCS parameters (like intensity, montage, duration, and repetition) on human cognition. This tDCS-iEEG-FPVS approach should provide effective technical guidelines to enhance a specific cognitive network.

### Functional Targeting and Specific Neuromodulation.

Through the analysis of frequency-tagged iEEG responses, we demonstrate that tDCS has an electrophysiological impact only on face-selective responses and not on general visual iEEG responses. This finding was made possible by a FPVS paradigm providing two distinct types of cognitive responses in the same experiment. The specificity of the neuromodulation induced by tDCS can be explained by the functional targeting of the VOTC, a region known to be critical for face processing. Since the FPVS task demands rapid (i.e., single glance) recognition of faces, the face-selective neural networks in the VOTC were more heavily engaged during the task.

This task-specific engagement of face-selective circuits likely made them more responsive to the effects of tDCS, which modulated their excitability in a task-dependent manner. In contrast, the broader visual networks involved in general visual processing were less engaged by this task, and therefore, less affected by the neuromodulation. Thus, the combined effects of functional targeting and increased neural engagement during face-selective processing could explain why tDCS specifically modulated face-selective responses with less influence on the general visual responses.

In the present study, a P10 (anode)—FT9 (return) tDCS montage was used specifically to target the right occipito-temporal cortex. The choice of the anodal P10 position was driven by previous scalp EEG studies that showed strong face-selective responses in this occipito-temporal scalp region using the same FPVS paradigm (20). Brain structures in the PTL of the VOTC were significantly impacted by tDCS but the largest amplitude neuromodulation was observed in the OCC, where face-selective activity (“occipital face area”) is typically localized according to the literature (39–42) and where a high EF magnitude was observed. The lack of significance in the OCC during and after tDCS is likely due to insufficient statistical power, as there were fewer iEEG contacts in this region (clinical constraints) compared to the ATL and PTL. Considering the same range and non-null-averaged EF values observed in our different ROI (0.14 to 0.40 V/m, *SI Appendix*, Table S5), the contribution of indirect neuromodulations [i.e., physiologically evoked neuromodulations in the highly connected VOTC; (43)] cannot be observed here. Indeed, to investigate this specific question, at least one ROI with significant iEEG responses and zero EF value would exist inside the brain network. In the present study, all ROI were under the influence (more or less strongly) of the induced EFs.

The small HD ring electrodes have the advantage of allowing a precise placement over the right occipito-temporal region without touching the iEEG electrodes that need to be protected (asepsis reasons). The right occipito-temporal cortex was targeted in this experiment because it holds one of the crucial nodes of the cortical face-selective network as shown with neuroimaging [EEG: (39), TMS: (40) and fMRI: (41)] and using direct intracerebral electrical stimulations (42). This original tDCS montage, with only two small HD ring electrodes, allows for significant neuromodulation. It is evidence showing that this montage can yield effective results. Usually, small ring electrodes are used in HD-tDCS with a montage of 4 × 1 i.e., one center-anode or -cathode and four return electrodes around. In the present study, it is interesting to note that HD electrodes can also be used in a “conventional” tDCS montage with only two electrodes.

Additionally, the small HD ring electrodes were certainly the key element to precisely target face-selective responses in the IOG with a sufficient EF. Computational studies with 4 × 1 HD montage demonstrated that small ring electrodes can focus and increase the intracerebral EF on the brain target compared to a bipolar sponge montage (44).

The face-selective electrophysiological neuromodulation is also crucial and important evidence of “functional targeting” that considers that cognitive brain networks already activated by a task become more sensitive to direct constant current stimulation (45, 46). An in vitro study showed that only synapses already experiencing plasticity would be influenced by DCS, while inactive synapses remained unmodulated (47). The dependence of tDCS on brain state has been mainly investigated using noninvasive neuroimaging [e.g.: scalp EEG (48) and fMRI (49)]. Our results provide the first evidence of functional targeting in humans using intracranial EEG recordings.

In conclusion, the current evidence provided by this intracerebral investigation in humans represents the first milestone demonstrating the ability of tDCS to induce objective, selective, and significant electrophysiological effects. Using this original method of simultaneous tDCS-iEEG-FPVS, future research could address important questions to define the most effective tDCS parameters for achieving the highest electrophysiological neuromodulation and, ultimately, sustainable behavioral changes.

## Materials and Methods

### Participants.

Eleven patients (six females; mean age: 31 ± 8 y) with focal drug-resistant epilepsy were prospectively included in this sham-controlled tDCS study. All patients provided written consent to take part in the study, which was conducted according to the principles expressed in the declaration of Helsinki and approved by a national ethics committee (Comité de Protection des Personnes Sud-Ouest et Outre Mer 4) certified by the French Ministry of Health (Institutional Review Board: IORG0009855). They were informed of the side effects of tDCS (tingling sensation, headache, etc.), and were instructed to report any discomfort during the experiment.

Their standard presurgical evaluation included neuropsychological tests and long-term (5-d period) high-resolution EEG video recordings combined with electrical source imaging analysis, positron emission tomography and high-resolution MRI. All participants had normal to corrected-to-normal vision and performed the Benton Face Recognition Test (50) before the iEEG exploration, with an average score of 43.3 ± 4.2 (10 patients had a Benton score above 39 of 54, reflecting normal performance in matching individual faces, and one patient had a score below 39 of 54, reflecting mild impairment). To identify the epileptogenic zone and the surrounding functional brain regions, patients were implanted with intracranial multicontact electrodes (iEEG) in the Epilepsy Unit of the University Hospital of Nancy. In all patients, iEEG allowed for the delineation of a single and spatially limited epileptogenic zone. Due to the right hemispheric dominance of face-perception, participants with at least one iEEG electrode implanted in the right hemisphere were included in this study.

### Transcranial Electrical Stimulation.

Before the tDCS experiment, a session of transcranial alternating current stimulation (0.5 mA, 2 min, 7 Hz, P10-FT9 montage) was performed to measure the EF in the different brain ROI. The EF was obtained by calculating the gradient of the voltage measured on each contact of a same iEEG electrode (for detailed information about the EF calculation, see ref. 8).

Sham-controlled anodal tDCS experiments were performed before the iEEG electrode removal (i.e., the last day of the iEEG investigation). This timing was decided to avoid tDCS aftereffects during the iEEG investigation which could influence the diagnosis (e.g., brain network instability). TDCS was performed using 2 HD-ring electrodes (12 mm external diameter; Soterix Medical®, New York, NY).

Both were inserted in an electrode-holder (24 mm diameter) filled with a conductive gel, creating a 4.52 cm^2^ stimulation area on the scalp for each electrode. The impedances were measured before tDCS stimulation and were always less than or equal to 5 kΩ during the active stimulation.

According to the HD electrodes’ geometry, the current densities generated on site were 0.44 mA.cm^−2^ for +2 mA intensity. The stimulation was delivered through these electrodes using a multichannel TES stimulator MxN-9 (Soterix Medical®, New York, NY). To target the VOTC, the anodal electrode was placed on the P10 scalp position and the common electrode on the FT9 scalp position (51).

**Fig. 5.**
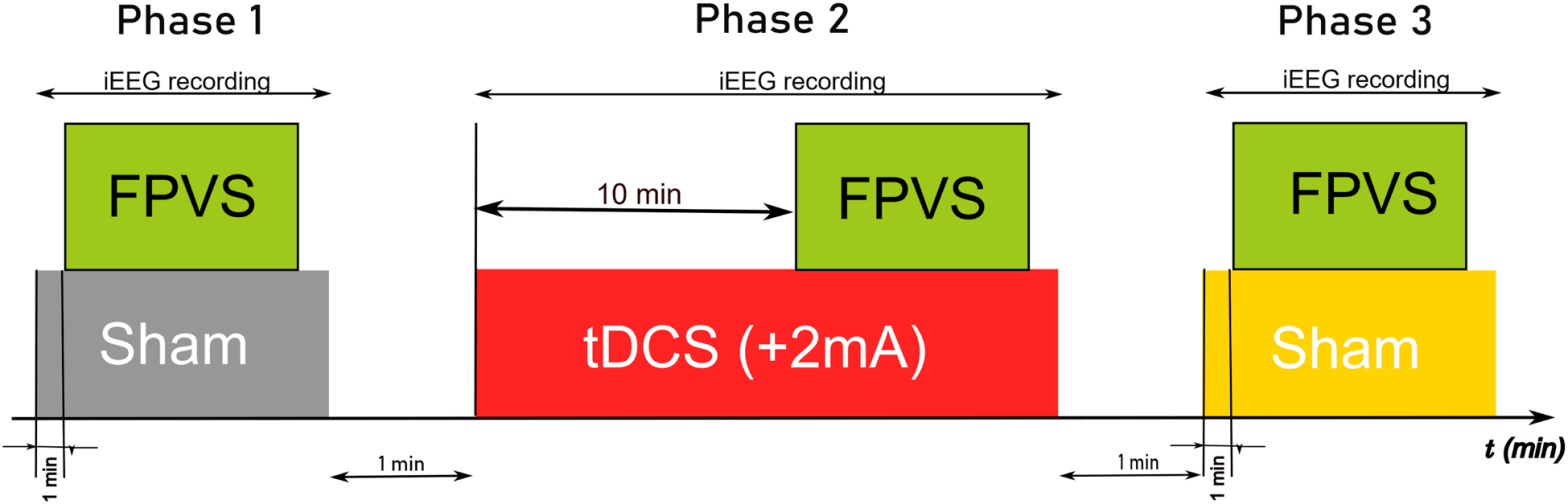
Design of the study protocol with tDCS, FPVS, and iEEG. During phase 1 (P1) and phase 3 (P3) a sham-tDCS session was performed during FPVS. Between them, phase 2 (P2) was performed with 10 min of active +2 mA tDCS alone, followed by 10 min of active tDCS during FPVS. IEEG recordings recorded brain activity during the three phases.

Two sham sessions were performed before (P1 phase) and after (P3 phase) tDCS in combination with FVPS (Fig. 5). During the sham stimulations, amplitude increased from 0 to 2 mA within the first 30 s, followed immediately by a decrease from 2 mA to 0 over the subsequent 30 s. The active anodal tDCS session (Phase 2) was performed at +2 mA (P10) for 20 min (with common parameters as outlined in ref. 3. Considering that it could take from 5 to 10 min of tDCS to produce persistent changes inside the brain, FPVS started in phase P2 after 10 min of active anodal tDCS (3, 52). Consequently, the P2 session consisted of 10 min of active tDCS alone, followed by 10 min of concurrent tDCS and FPVS.

### FPVS.

#### Stimuli.

200 natural images of various nonface objects from 14 nonface categories were used (cats, dogs, vegetables, horses, flowers, birds, fruits, phones...) in addition to 50 natural images of human faces. Each image showcased an unsegmented object or face near the center, varying in size, viewpoint, lighting, and background. All images were standardized for mean pixel luminance and contrast. The same stimuli were used by ref. 21 to find the face-selective ventral occipito-temporal map of the human brain with intracerebral potentials.

#### Procedure.

**Fig. 6.**
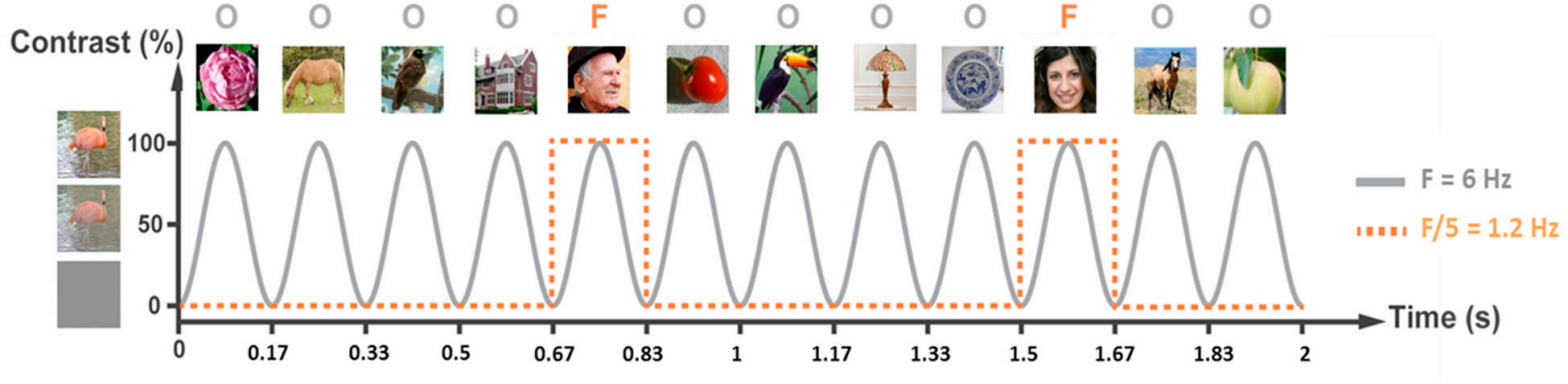
FPVS used in this tDCS-iEEG experiment. Images of objects were presented by sinusoidal contrast modulation at a rate of six stimuli per second (6 Hz). In the periodic condition shown here, a different face image was presented every five stimuli (i.e., appearing at the frequency of 6/5 = 1.2 Hz). This stimulation sequence recruits ventral occipito-temporal brain regions and therefore obtains iEEG general visual and face-selective evoked responses.

Participants were shown continuous sequences with highly variable natural images of objects at a frequency of 6 Hz through sinusoidal contrast modulation. Highly variable natural images of faces presented periodically were presented every fifth image (i.e., at 1.2 Hz = 6/5 Hz) (Fig. 6). Participants were unaware of the periodicity of the faces. A sequence lasted 79 s, including 75 s of stimulation (i.e., 90 images of human face and 360 images of natural objects) at full contrast flanked by 2 s of fade-in and 2 s of fade-out, where contrast gradually increased or decreased, respectively. This extended duration of the sequence results in a high-frequency resolution (i.e., 71 s so 0.0141 Hz), enabling the isolation of the specific response within a narrow frequency range, significantly improving its SNR. The FPVS paradigm was repeated two times during each phase (i.e., 2 sessions * 3 phases = 6 sessions in all). Throughout the sequences, participants were directed to maintain their gaze on a persistent small blue cross positioned at the center of the stimuli. Their task was only to identify brief color changes (from blue to red) in this central fixation cross, lasting 500 ms.

### Intracranial EEG.

#### Recording.

For each patient, an individual stereotactic implantation plan was defined according to the presurgical evaluation and epileptogenic zone localization hypotheses. Under general anesthesia, the iEEG electrodes (0.8 mm-diameter, from 5 to 18 Platinum/Iridium contacts with 2 mm length separated by 1.5 mm insulator; Dixi Medical®, Besançon, France) were implanted according to a standard stereotactic procedure (53). The iEEG electrodes were inserted through a screw (2.45 mm-diameter) and secured with a tight seal to prevent cerebrospinal fluid leak. Immediately after the implantation, patients underwent a postoperative CT-scan.

Using a coregistration of the CT-scan with the preoperation high-resolution MRI (voxel-based registration; SPM 8 toolbox for Matlab; MathWorks, Natick, MA), the anatomical positions of each electrode’s contacts were localized, and potential surgical complications were inspected. Due to the minimally invasive procedure used for iEEG investigation, anatomical structures were not displaced in the intracranial volume (no cerebrospinal fluid leakage and no brain swelling) and MRI-Computed Tomography coregistration can be assumed as very precise (<1 mm). IEEG signals were recorded with a 256-channel (2 × 128 channels) amplifier with a 1 kHz sampling rate (LTM256, Micromed, Mogliano, Italy). The Fpz scalp electrode was set as the reference electrode for the recording and a bipolar montage was then computed for each patient. Among 947 iEEG contacts, 67 iEEG contacts showed saturated amplitude during tDCS (Phase P2) so that they were excluded from the analysis. No significant general visual or face-selective iEEG responses were found in these contacts before the tDCS, so their exclusion did not change the results.

#### Anatomical localization.

The 3D position of each iEEG contact was automatically detected in the individual CT-scan [60] and visually defined using patients’ MRI-CT coregistration. Each individual brain was subdivided into different brain ROI using individual anatomical landmarks (i.e., gyri and sulci) instead of normalizing all brains which can blur the individuality of anatomical organization. IEEG contacts were then localized according to these brain ROI and grouped by anatomical localization across all participants. The VOTC was parcellated according to the atlas of Kim et al. (54). The VOTC sulci were used as mesio-lateral landmarks (especially collateral sulcus and occipito-temporal sulcus) and MR coronal planes gave landmarks for antero-posterior brain structures.

#### Spectral analysis.

**Fig. 7.**
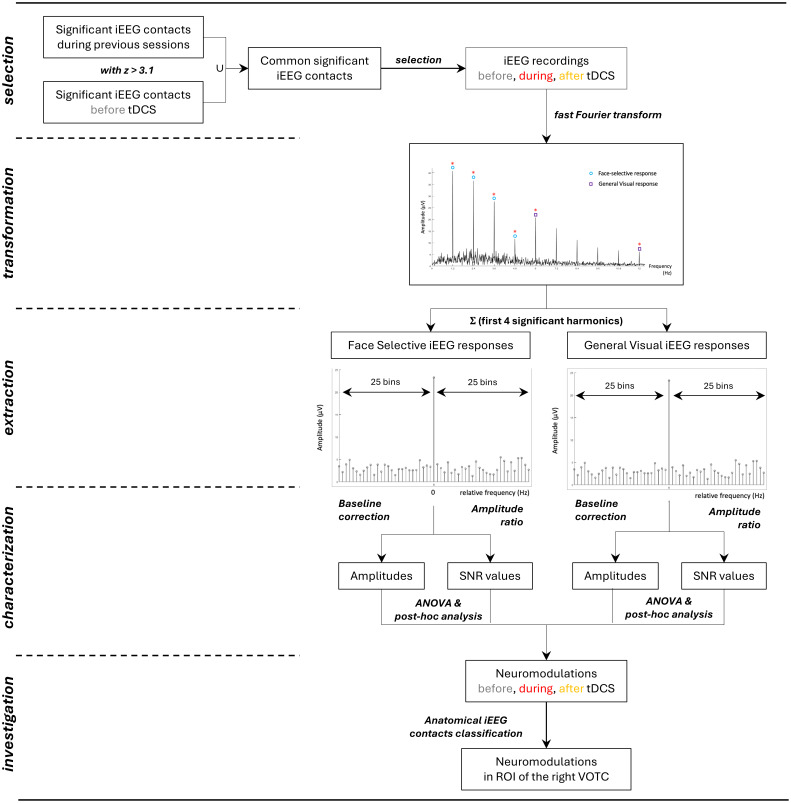
Overview of the iEEG data analysis. Raw iEEG signals were analyzed in the frequency domain after FFT. A baseline correction was applied to extract the iEEG response amplitudes. SNR was also estimated. Then, ANOVA tests with post hoc comparisons were used to compare the results before, during, and after the tDCS and across the different ROI.

Data segments of iEEG signals corresponding to the FPVS sequences were selected (79-s segments, −2 to +81 s) from the recordings. In our main data analyses, we refrained from conducting artifact rejection procedures since intracerebral artifacts, primarily stemming from epileptic spikes, along with electro-oculographic and electromyographic activities (attributable to our use of a prefrontal scalp electrode as the reference electrode for most patients), exhibit a more extensive distribution across the frequency spectrum compared to the specific frequencies of interest: 1.2 and 6 Hz, and their corresponding harmonics. The 79 s data segments were cropped to contain an integer number of 1.2-Hz cycles beginning 2 s after the onset of the sequence (right at the end of the fade-in period) until ∼77 s, before stimulus fade-out (90 face cycles ∼75 s). Sequences were averaged in the time domain separately for each participant. A fast Fourier transform (FFT) was applied on these averaged segments (see all raw amplitude spectrum in *SI Appendix*, Fig. S4), then the amplitude spectra were extracted for all intracerebral contacts (Fig. 7).

#### Extraction of iEEG evoked responses.

The FPVS sessions applied in this study allows us to identify and separate two different types of iEEG responses: i) a general visual iEEG response occurring at the base stimulation frequency (6 Hz) and its harmonics, as well as ii) a face-selective iEEG response at 1.2 Hz and its harmonics. Significant face- selective responses at the face stimulation frequency (1.2 Hz) and its harmonics (2.4, 3.6 Hz, etc.) were detected by transforming the frequency spectra to z scores. The z scores were computed as the difference between amplitude at each frequency bin and the mean amplitude of the corresponding 48 surrounding bins (25 bins on each side, i.e., 50 bins, but excluding the two bins directly adjacent to the bin of interest, i.e., 48 bins) divided by the SD of amplitudes in the corresponding 48 surrounding bins. A contact was considered as face-selective if the z score of the iEEG response was >3.1 (i.e., *P* < 0.001, one-tailed: signal > noise) for at least one of the first four face-selective frequency harmonics in the periodic condition (1.2, 2.4, 3.6, or 4.8 Hz). The same procedure was performed to extract significant general visual responses at 6 Hz and its harmonics (18, 24, 30 Hz) (Fig. 7).

#### Characterization of iEEG evoked responses.

Baseline-corrected amplitudes were calculated as the difference between the amplitude at each frequency bin and the mean of 48 corresponding surrounding bins (25 bins on each side, i.e., 50 bins, but excluding the two bins directly adjacent to the bin of interest, so, 48 bins). The general visual and the face-selective iEEG responses were then quantified at each iEEG contact as the sum of the baseline-subtracted amplitude across harmonics. Face-selective responses were quantified as the sum of the baseline-subtracted amplitudes at the face-selective frequency harmonics from the 1st until the 4th (1.2 until 4.8 Hz). General visual responses were similarly quantified by summing the amplitudes from the 1st until the 4th base frequency harmonics (six until 24 Hz). Therefore, for each iEEG contact, we obtained two amplitude values that respectively represented the overall face-selective response and the overall general visual response during each of the three phases (before, during, and after tDCS) for the condition tested. Finally, SNR spectra were calculated as the ratio between the amplitude at each frequency bin and the average of the corresponding 48 surrounding bins (Fig. 7).

### Intracranial Neuromodulation Investigation.

#### Selection of iEEG contacts.

To compare the iEEG neuromodulation before, during, and after tDCS, selective iEEG contacts (i.e., contacts that recorded iEEG evoked responses for either general or face-selective responses) were selected. To do so, during the 2 d leading up to the tDCS experiment (D-2 and D-1), two sessions of FPVS were performed without tDCS using the same paradigm as the one used in this study. The analysis of the iEEG signals during D-2 and D-1 led to the identification of a set of iEEG contacts in the right hemisphere that exhibited statistically significant general visual and face-selective responses. Due to the robustness of the FPVS approach in this protocol (20), this set of iEEG contacts was assigned to our study to compute the mean and SEM of the amplitudes and the SNR during the three phases (Fig. 7).

#### Neuromodulation according to the time phases.

To characterize the tDCS effect over time, means and SEM baseline-corrected amplitudes and SNR of iEEG evoked responses recorded in all the contacts in the right hemisphere, and then in the VOTC. These calculations were performed for both face-selective and general visual responses during P1, P2, and P3 phases. Repeated measure ANOVA tests with post hoc comparisons were performed with the null hypothesis that tDCS has no effect on the iEEG evoked responses [i.e., no baseline-corrected amplitude (or SNR) differences between the iEEG responses] for both general and face-selective responses across the different phases of the experiment (alpha risk: 0.05) (Fig. 7).

#### Neuromodulation according to the anatomy.

To investigate the tDCS effect on the different regions of the VTOC and therefore investigate the spatial targeting of the small ring electrodes, the VOTC was divided into three brain ROI using the anatomo-functional classification from ref. 21: ATL, anterior temporal lobe; PTL, posterior temporal lobe; OCC, occipital lobe. Means and SEM baseline-corrected amplitudes and SNR of iEEG evoked responses recorded in the contacts of the different ROI. Statistical comparisons were performed using repeated measure ANOVA tests with post hoc comparisons with the null hypothesis that intracranial neuromodulation will be the same in all ROI (Fig. 7).

## Supplementary Material

Appendix 01 (PDF)

## Data Availability

Anonymized iEEG data (amplitude and SNR values) data have been deposited in (55).
