# Supplementary material for: Transcranial direct current stimulation neuromodulates intracranial cognitive evoked activity in humans
Source: Proc Natl Acad Sci U S A. 2025 Jun 3;122(23):e2416541122. doi: 10.1073/pnas.2416541122 (PMC12168024; doi:10.1073/pnas.2416541122)
Supplement: Supplementary file 1 — Appendix 01 (PDF) [file pnas.2416541122.sapp.pdf]

Supporting Information for

Transcranial Direct Current Stimulation Neuromodulates Intracranial Cognitive Evoked Potentials in Human

Mireille Tabikh [a], Tom Quetu [a], Louis Maillard [a, b],
Sophie Colnat-Coulbois [a, c], Bruno Rossion [a, b], Laurent Koessler [a,d]

[a] *IMoPA, UMR7365, CNRS and University of Lorraine, Nancy, F-54000, France*

[b] *Neurology Unit, University Hospital of Nancy, Nancy, F-54000 France*

[c] *Neurosurgery Unit, University Hospital of Nancy, Nancy, F-54000 France*

[d] *Bioserenity, Paris, F-75013 France*

Laurent Koessler, 29 Avenue of Maréchal de Lattre de Tassigny, Central Hospital, Krug Pavilion, 1st Floor, Nancy, F-54000, France; email: laurent.koessler@cnrs.fr

This PDF file includes:

Figures S1 to S4
Legends for Figures S1 to S4
Table S5

Supporting Information Figures

Fig. S1.

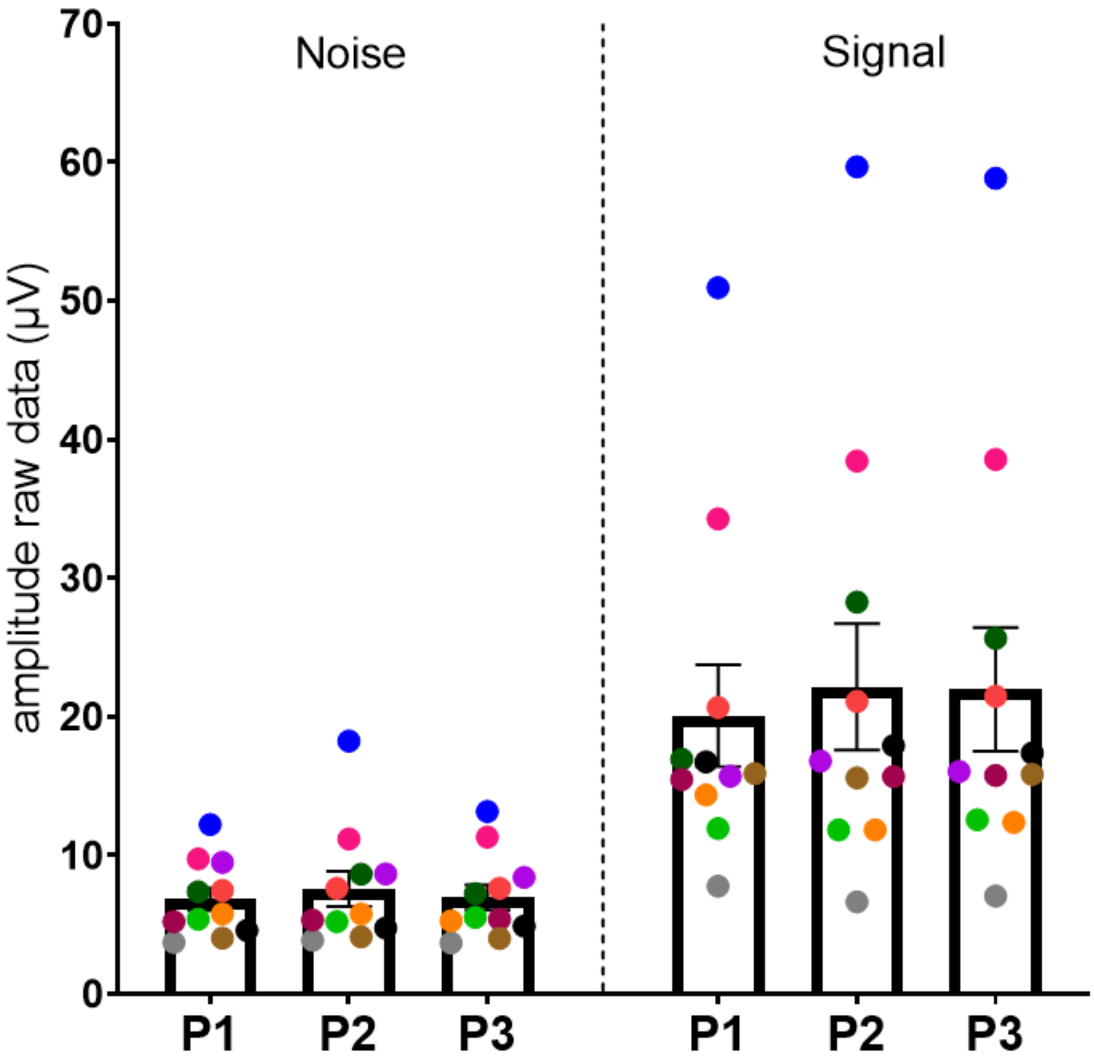

Figure S1: Average amplitudes of the raw iEEG signals (i.e., without baseline correction) for both signals (i.e., the face-selective responses at 1.2Hz and harmonics) and noise (i.e., the surrounding frequency bands around face-selective responses) before (P1), during (P2) and after tDCS. No noise amplitude increase, and weak amplitude value (by comparison to signal) were observed in our cohort during tDCS. Each dot represents the average amplitudes of iEEG signals for each patient.

Fig. S2.

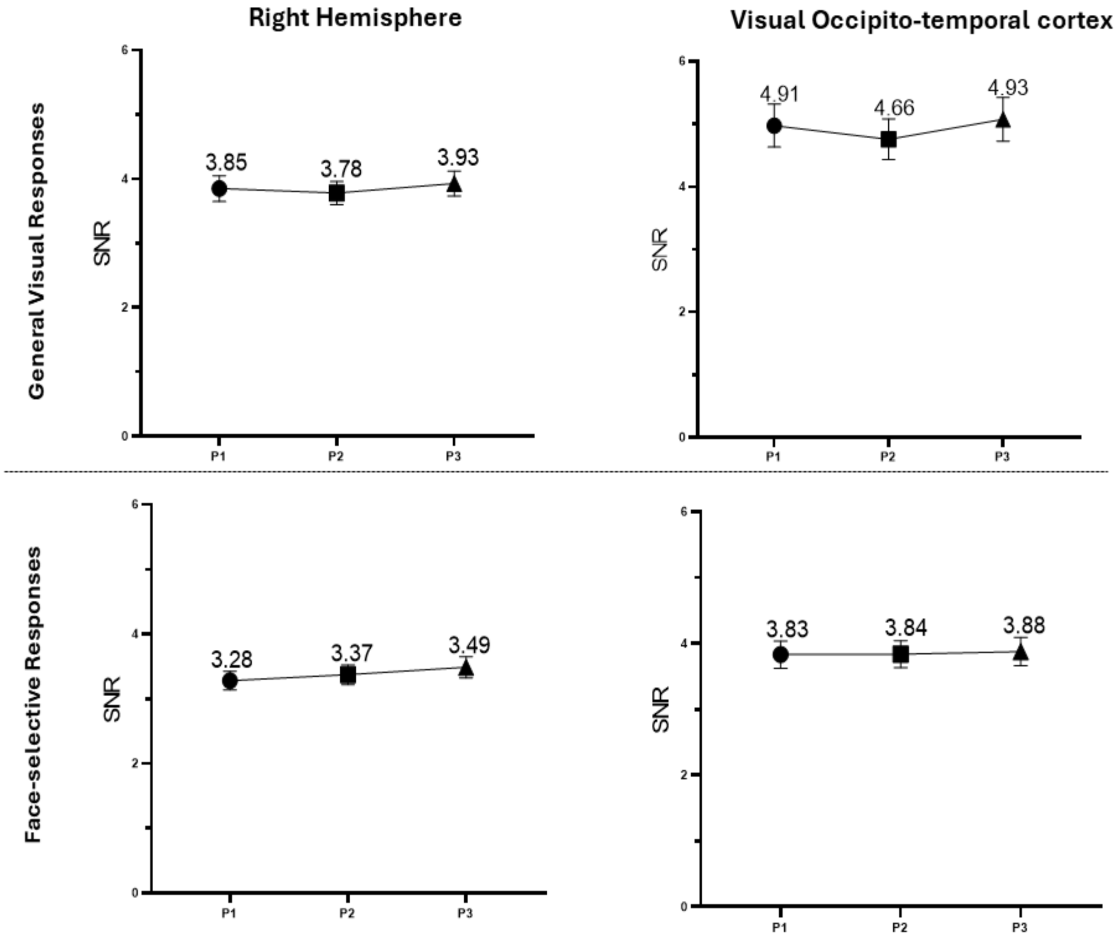

Figure S2: Neuromodulation of iEEG general visual (on the top) and face-selective (on the bottom) responses across the three successive phases of the tDCS experiment (before, P1; during, P2; after, P3) in the right hemisphere (left column) and in the right visual occipito-temporal cortex (right column). Averaged Signal to Noise Ratios (SNR) and standard errors of the mean (SEM) of the iEEG evoked responses were computed during the three different phases for the eleven patients. In the right hemisphere, 260 and 302 iEEG contacts were used to compute the average baseline-corrected amplitudes whereas 147 and 181 contacts were selected for the general visual responses and the face-selective responses, respectively. No significant neuromodulation was observed for both responses ($p>0.5$) between the three different phases (P1, P2, P3).
Phases: ● P1 (sham, before tDCS), ■ P2 (active, during tDCS), ▲ P3 (sham, after tDCS).

Fig. S3.

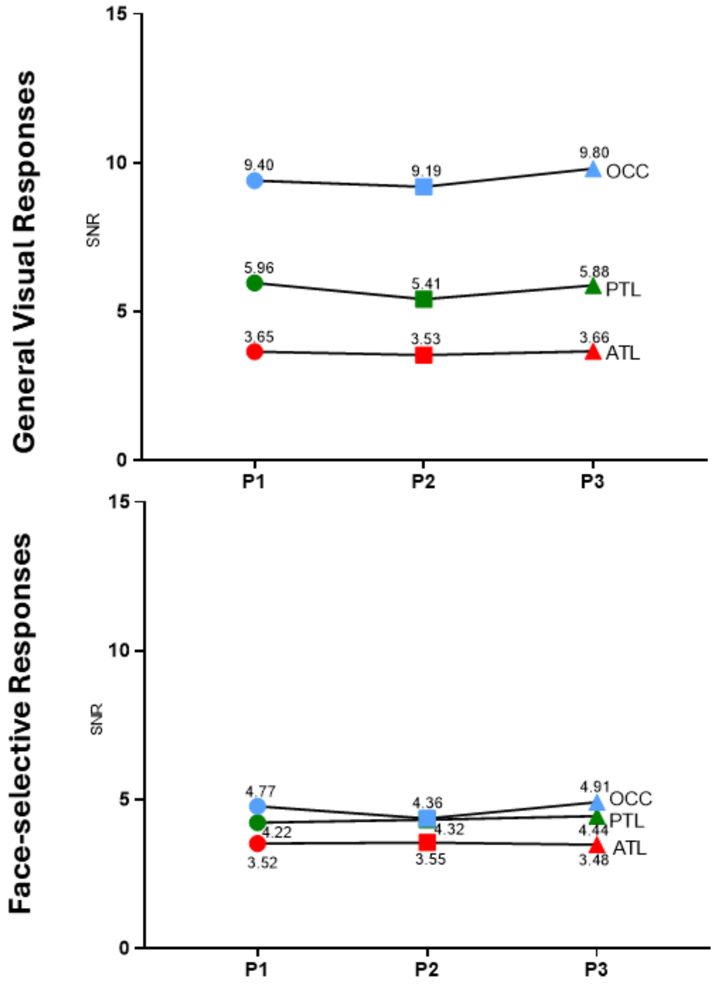

Figure S3: Neuromodulation of iEEG general visual and face-selective in the right ventral occipito-temporal cortex (VOTC). SNRs of the general visual and the face-selective iEEG responses in the three ROIs during the three phases. No significant difference was found in any of the 3 regions in the VOTC for both types of responses between the three different phases (P1, P2, P3). VOTC: ventral occipito-temporal cortex, ATL: Anterior Temporal Lobe, PTL: Posterior Temporal Lobe, OCC: Occipital Lobe. ● P1(sham, before tDCS), ■ P2(active, during tDCS), and ▲ P3 (sham, after tDCS).

Fig. S4.

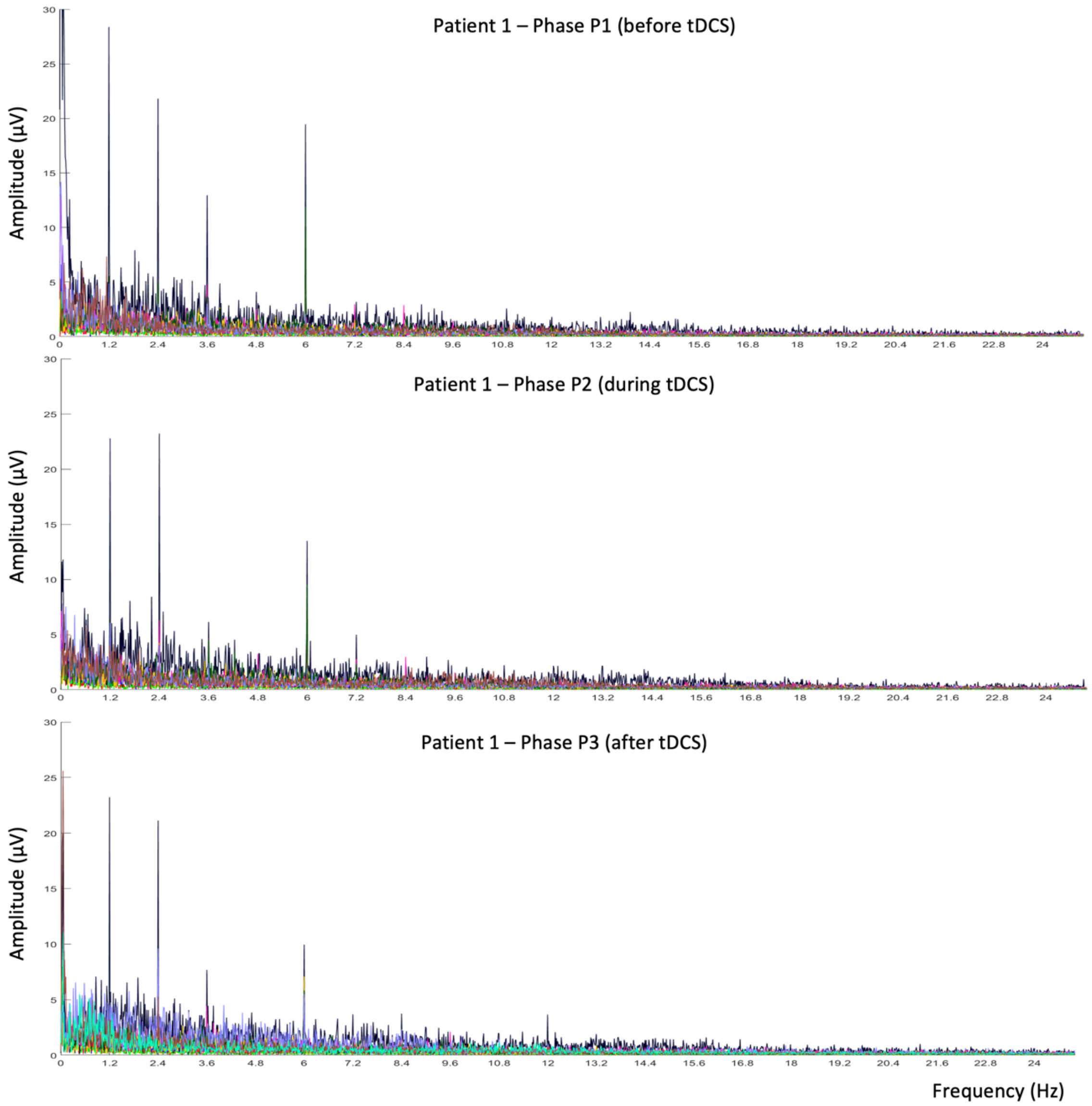
Patient 1 – Phase P1 (before tDCS)
Patient 1 – Phase P2 (during tDCS)
Patient 1 – Phase P3 (after tDCS)
Amplitude (µV)
Amplitude (µV)
Amplitude (µV)
Frequency (Hz)

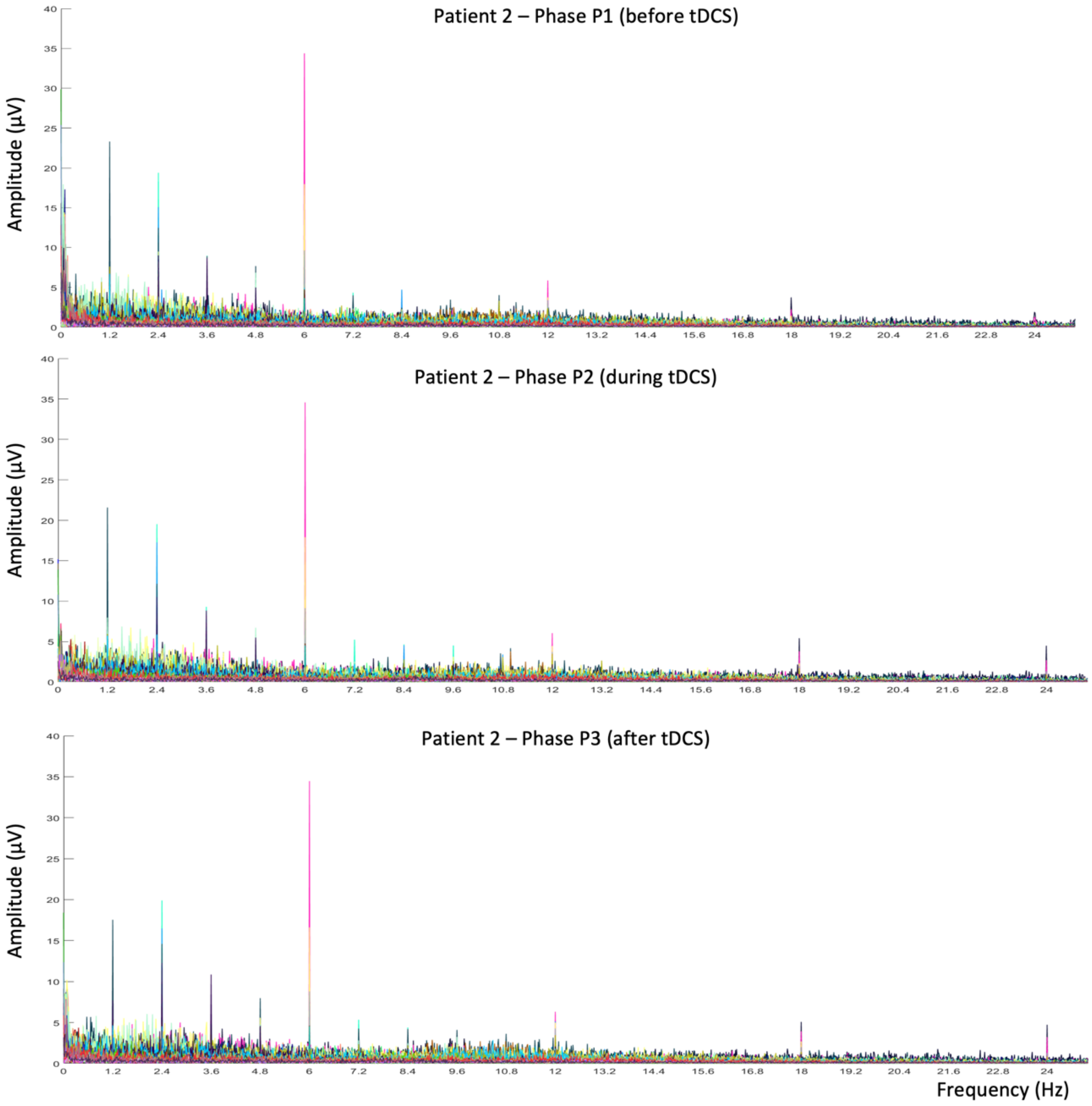
Patient 2 – Phase P1 (before tDCS)
Amplitude (µV)
0 5 10 15 20 25 30 35 40
0 1.2 2.4 3.6 4.8 6 7.2 8.4 9.6 10.8 12 13.2 14.4 15.6 16.8 18 19.2 20.4 21.6 22.8 24
Patient 2 – Phase P2 (during tDCS)
Amplitude (µV)
0 5 10 15 20 25 30 35 40
0 1.2 2.4 3.6 4.8 6 7.2 8.4 9.6 10.8 12 13.2 14.4 15.6 16.8 18 19.2 20.4 21.6 22.8 24
Patient 2 – Phase P3 (after tDCS)
Amplitude (µV)
0 5 10 15 20 25 30 35 40
0 1.2 2.4 3.6 4.8 6 7.2 8.4 9.6 10.8 12 13.2 14.4 15.6 16.8 18 19.2 20.4 21.6 22.8 24
Frequency (Hz)

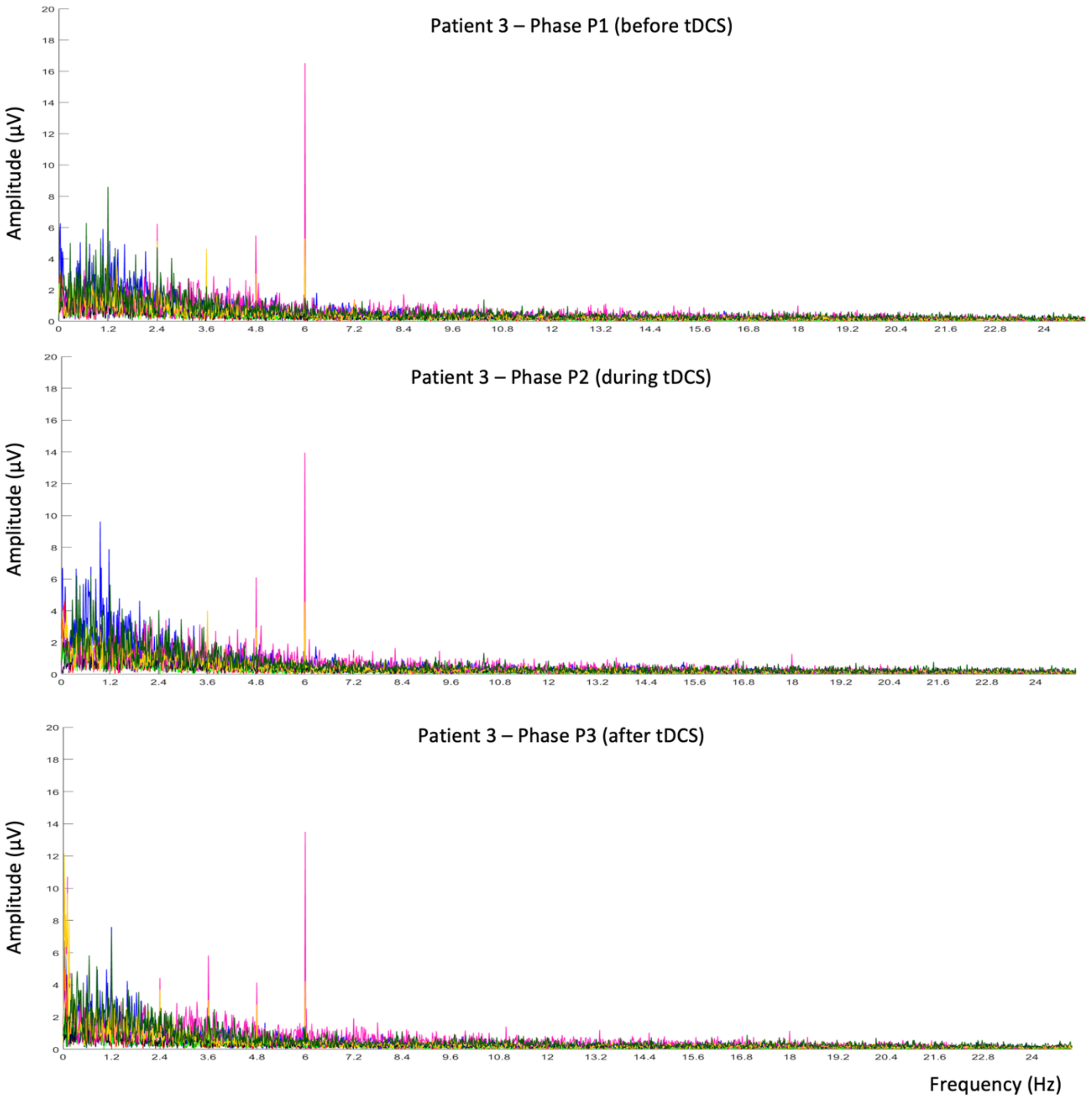

Patient 3 – Phase P1 (before tDCS)
Amplitude (µV)
Patient 3 – Phase P2 (during tDCS)
Amplitude (µV)
Patient 3 – Phase P3 (after tDCS)
Amplitude (µV)
Frequency (Hz)

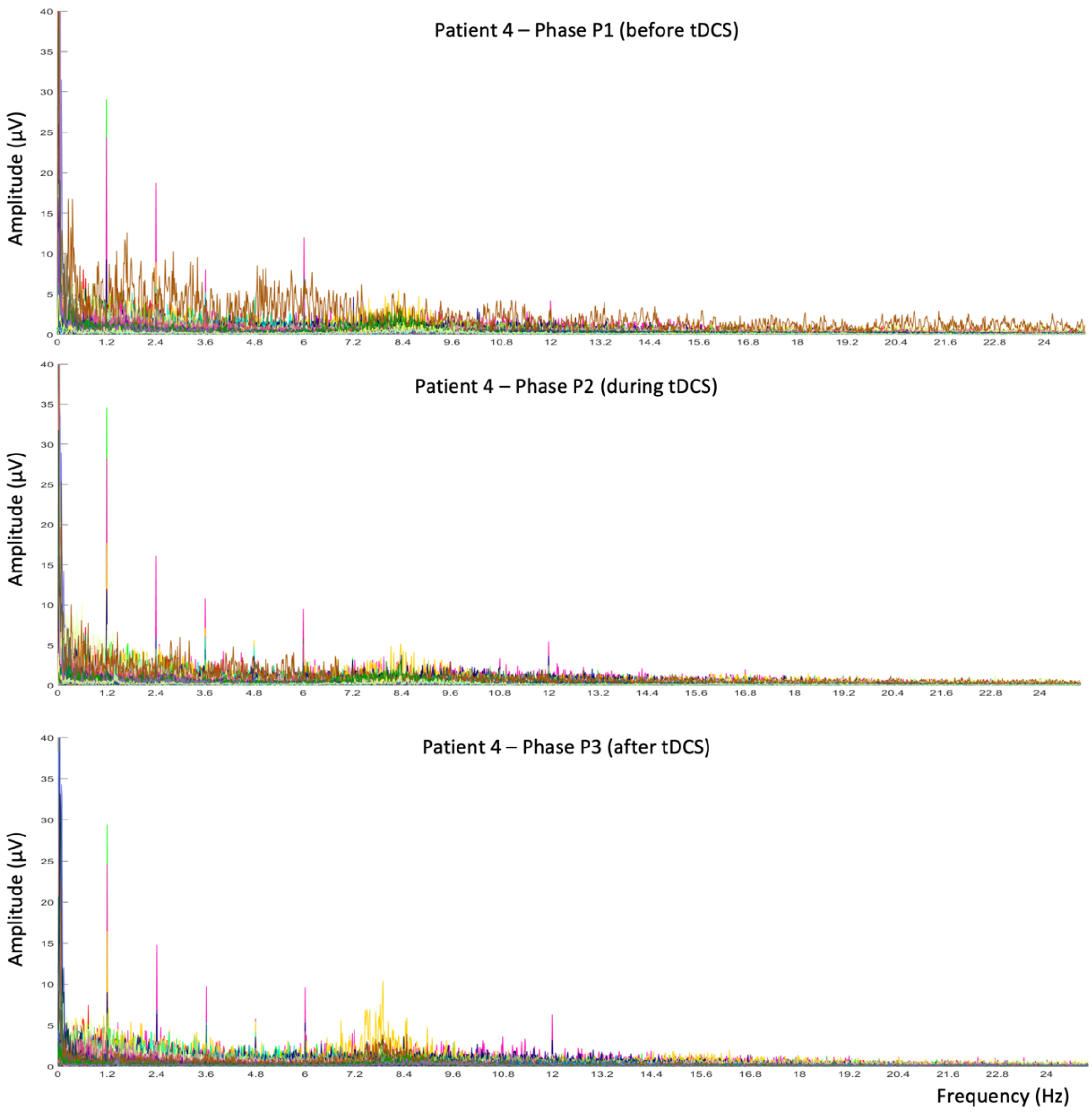
Patient 4 – Phase P1 (before tDCS)
Amplitude (µV)
Patient 4 – Phase P2 (during tDCS)
Amplitude (µV)
Patient 4 – Phase P3 (after tDCS)
Amplitude (µV)
Frequency (Hz)

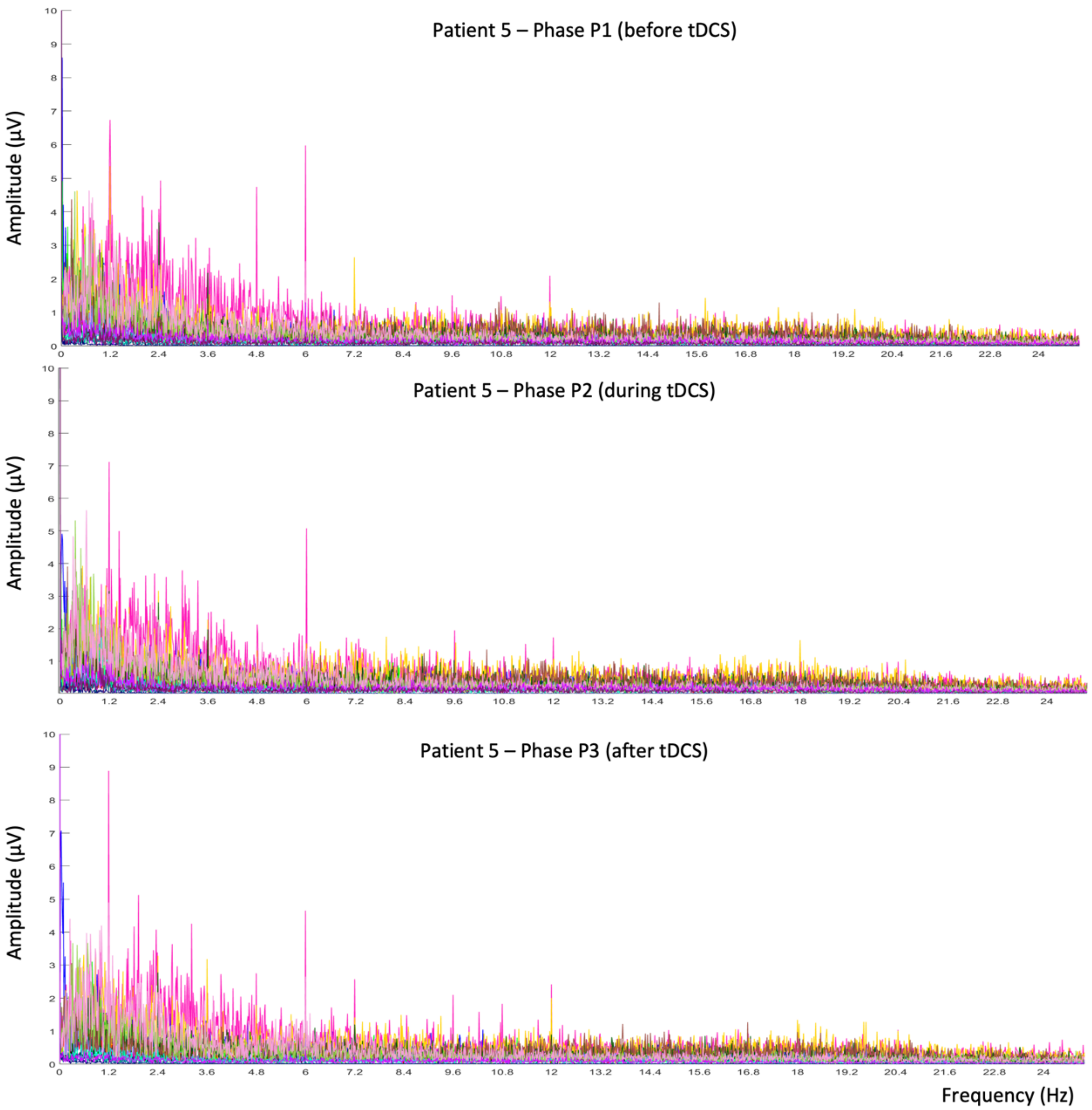
Patient 5 – Phase P1 (before tDCS)
Patient 5 – Phase P2 (during tDCS)
Patient 5 – Phase P3 (after tDCS)
Amplitude (μV)
Frequency (Hz)
0 1.2 2.4 3.6 4.8 6 7.2 8.4 9.6 10.8 12 13.2 14.4 15.6 16.8 18 19.2 20.4 21.6 22.8 24
0 1 2 3 4 5 6 7 8 9 10

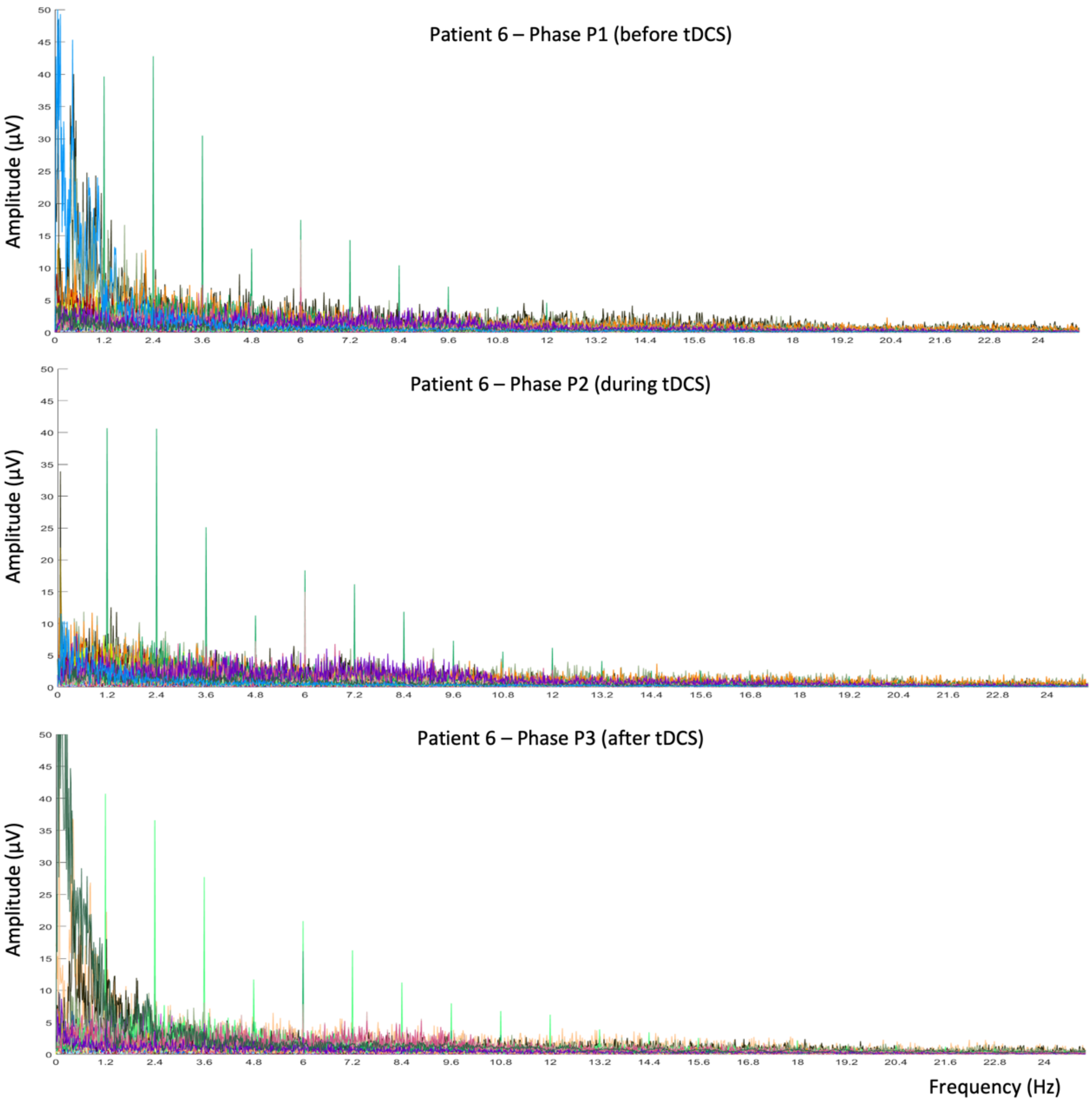
Patient 6 – Phase P1 (before tDCS)
Patient 6 – Phase P2 (during tDCS)
Patient 6 – Phase P3 (after tDCS)
Amplitude (µV)
Frequency (Hz)

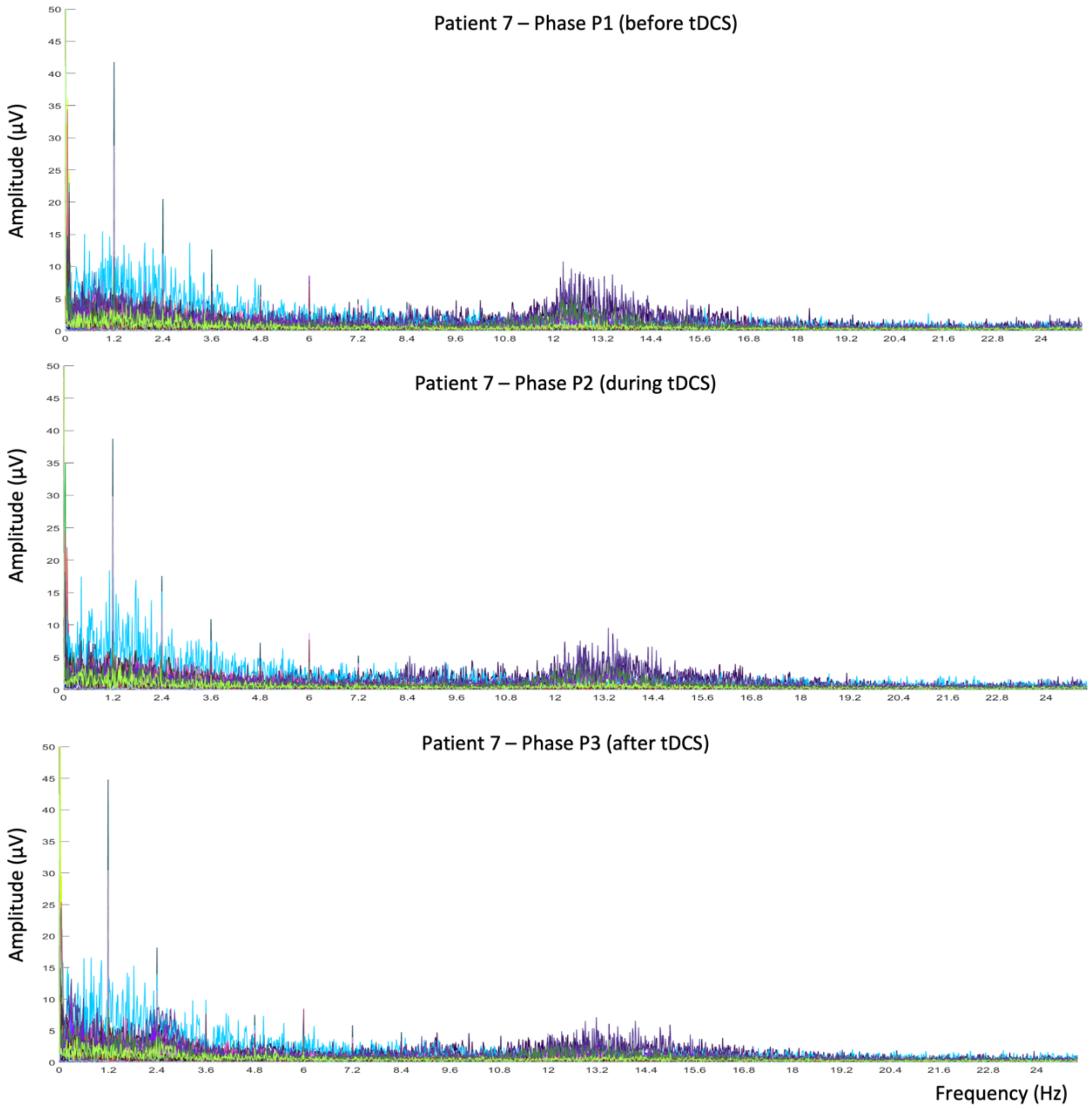
Patient 7 – Phase P1 (before tDCS)
Amplitude (µV)
Patient 7 – Phase P2 (during tDCS)
Amplitude (µV)
Patient 7 – Phase P3 (after tDCS)
Amplitude (µV)
0
1.2
2.4
3.6
4.8
6
7.2
8.4
9.6
10.8
12
13.2
14.4
15.6
16.8
18
19.2
20.4
21.6
22.8
24
0
5
10
15
20
25
30
35
40
45
50
Frequency (Hz)

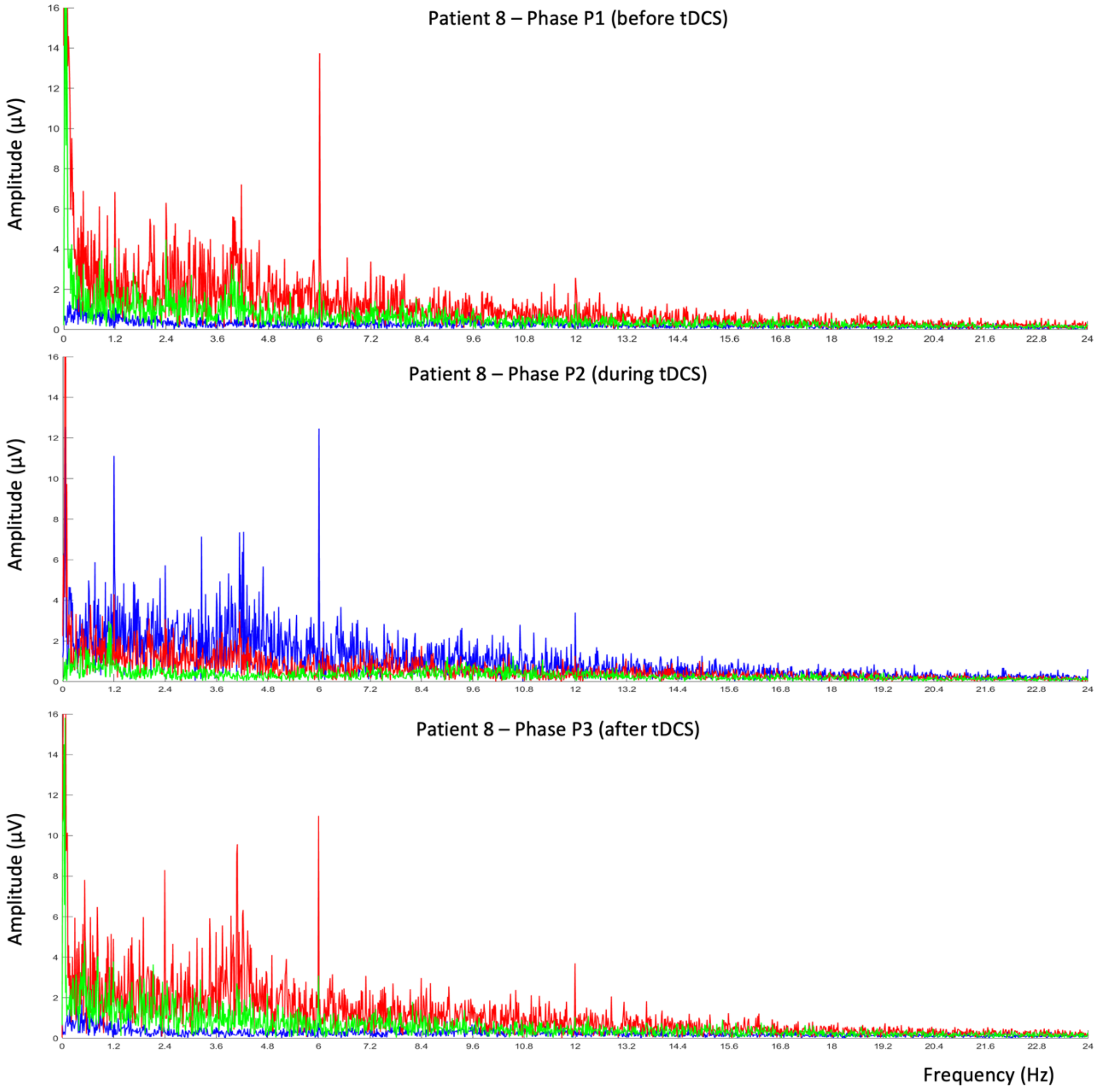

Patient 8 – Phase P1 (before tDCS)
Amplitude (µV)
Patient 8 – Phase P2 (during tDCS)
Amplitude (µV)
Patient 8 – Phase P3 (after tDCS)
Amplitude (µV)
Frequency (Hz)

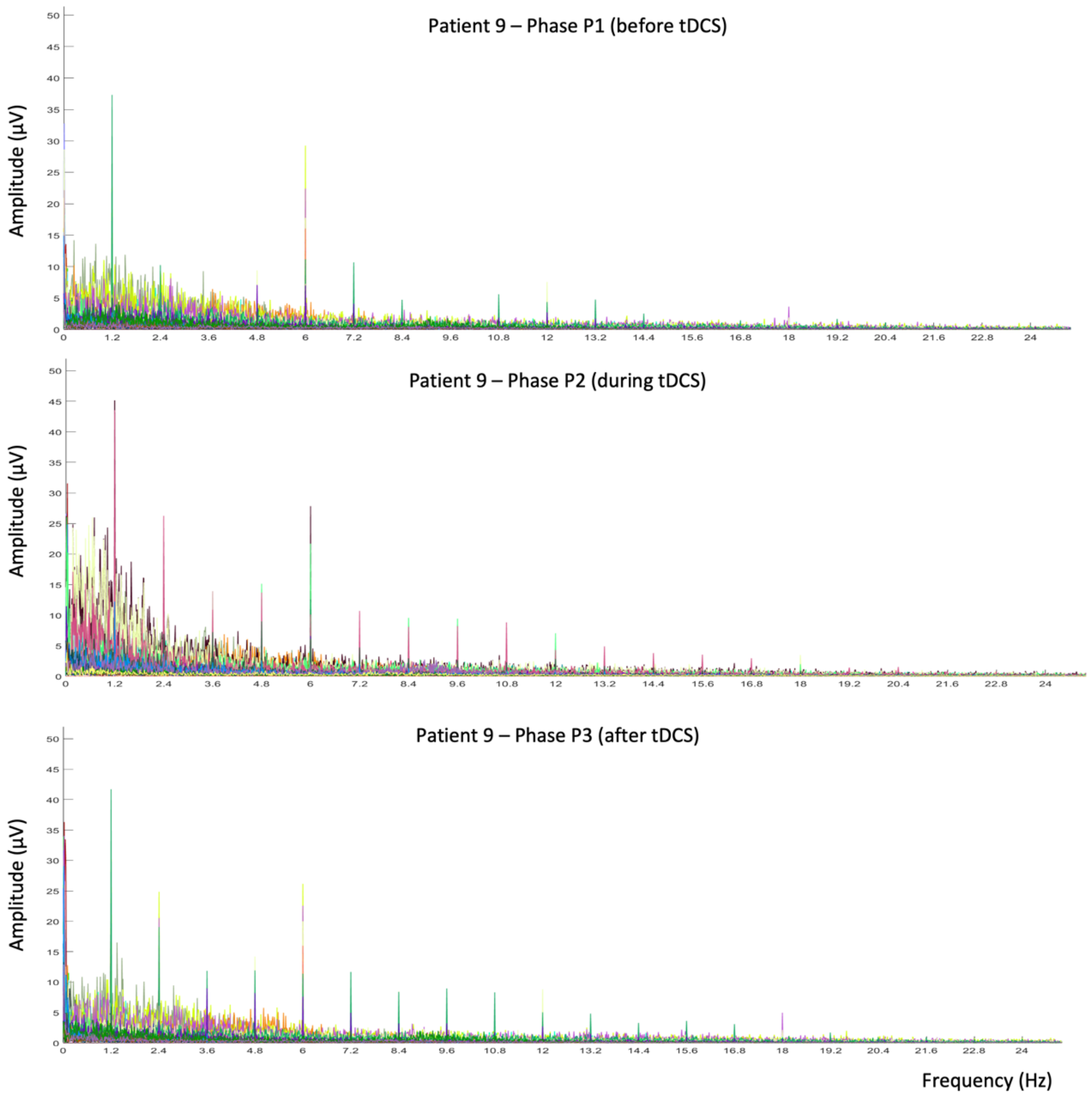

Patient 9 – Phase P1 (before tDCS)
Amplitude (µV)
Patient 9 – Phase P2 (during tDCS)
Amplitude (µV)
Patient 9 – Phase P3 (after tDCS)
Amplitude (µV)
Frequency (Hz)

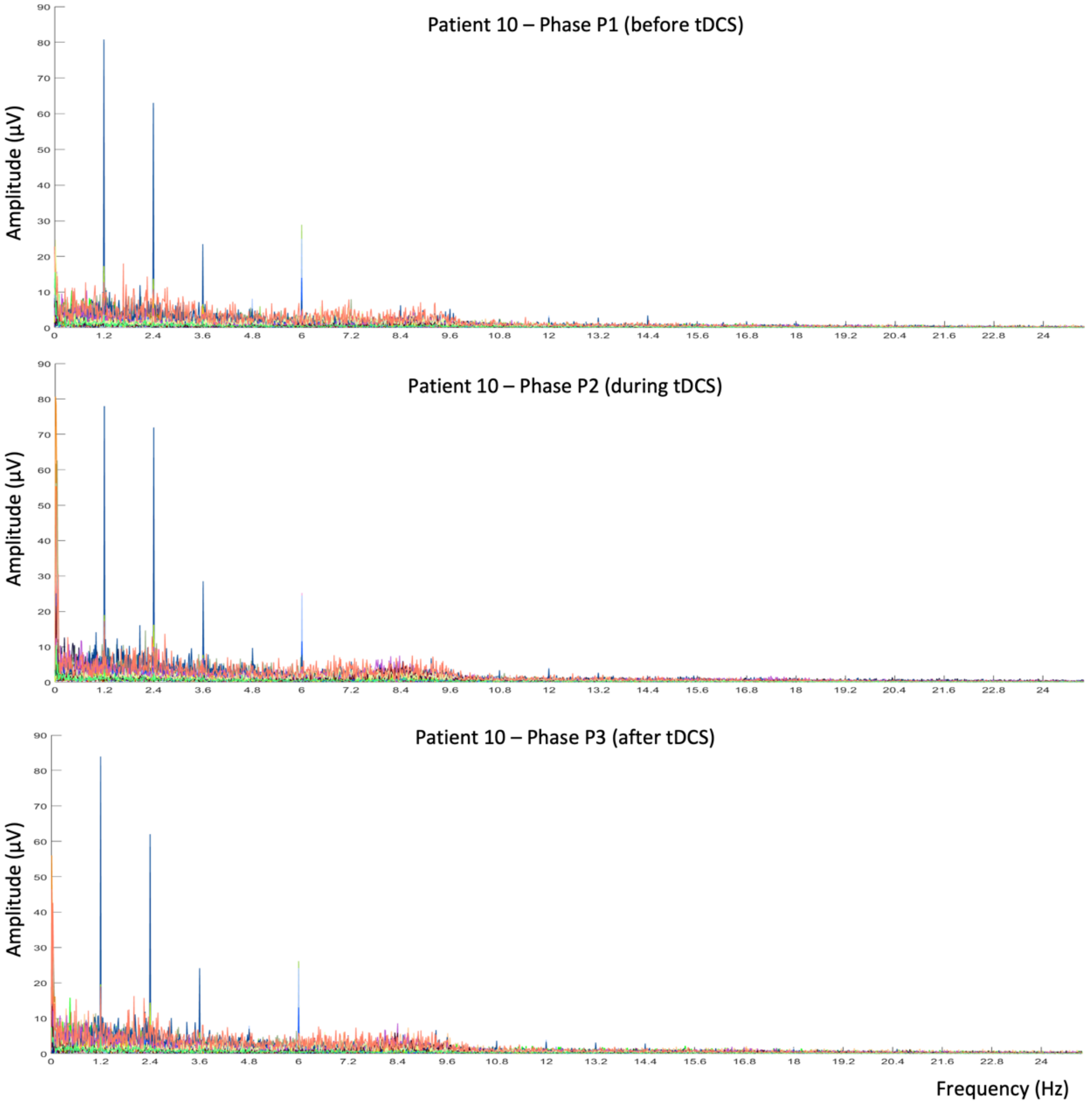
Patient 10 – Phase P1 (before tDCS)
Amplitude (µV)
Patient 10 – Phase P2 (during tDCS)
Amplitude (µV)
Patient 10 – Phase P3 (after tDCS)
Amplitude (µV)
Frequency (Hz)

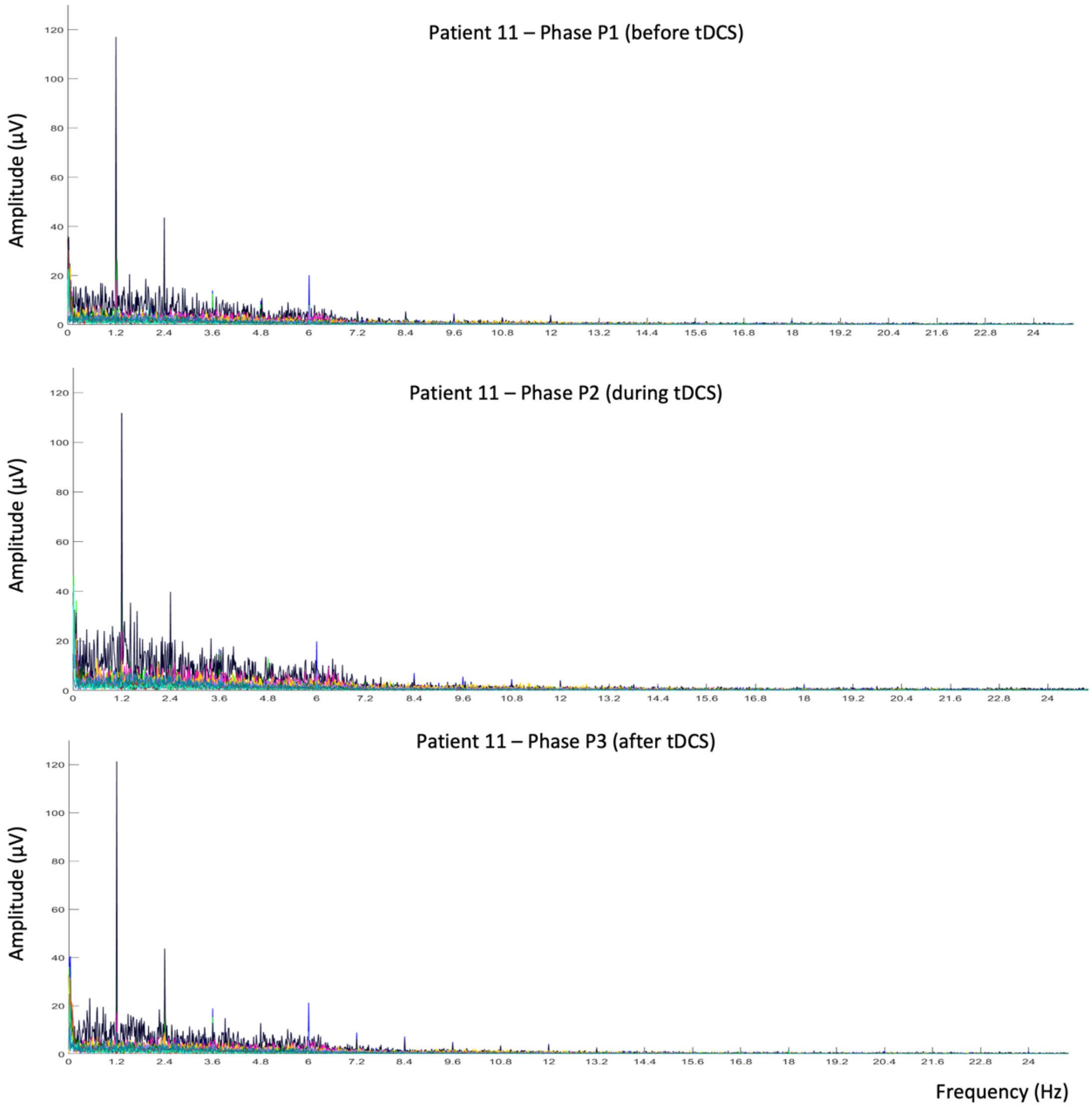

Figure S4: Raw amplitude spectra for all patients in all significant iEEG contacts (for both general visual and face-selective responses) before (P1), during (P2) and after (P3) tDCS. Note that no important non-physiological artifact due to tDCS appeared and that the amplitude range remains stable during the three phases in each patient.

Table. S5 Mean electric field (EF) magnitudes in the different brain structures

	ATL	Mean EF values (V/m)	N contacts
shallowest	antMTG	0.40	23
↓	antOTS	0.17	36
	antCOS	0.18	33
deepest	antFG	0.15	13

	PTL	Mean EF values (V/m)	N contacts
shallowest	MTG/ITG	0.23	16
↓	LatFG	0.17	16
deepest	MFG	0.14	21

	OCC	Mean EF values (V/m)	N contacts
shallowest	IOG	0.38	11
deepest	VMO	0.14	6

Table. S5 Mean electric field (EF) magnitudes in the different brain structures

ATL: anterior temporal lobe, PTL: posterior temporal lobe, OCC: occipital lobe, MTG: middle temporal gyrus, OTS: occipito-temporal sulcus, COS: collateral sulcus, FG: fusiform gyrus, ITG: inferior temporal gyrus, LFG: lateral fusiform gyrus, IOG: inferior occipital gyrus, VMO: ventromedial occipital
